# *Medicago truncatula* genotype drives the plant nutritional strategy and its associated rhizosphere bacterial communities

**DOI:** 10.1111/nph.20272

**Published:** 2024-11-28

**Authors:** Anouk Zancarini, Christine Le Signor, Sébastien Terrat, Julie Aubert, Christophe Salon, Nathalie Munier‐Jolain, Christophe Mougel

**Affiliations:** ^1^ IGEPP, INRAE, Institut Agro Univ Rennes 35653 Le Rheu France; ^2^ Agroécologie, INRAE, Institut Agro, Univ. Bourgogne, Univ. Bourgogne Franche‐Comté F‐21000 Dijon France; ^3^ Université Paris‐Saclay, AgroParisTech, INRAE, UMR MIA Paris‐Saclay 91120 Palaiseau France

**Keywords:** bacterial communities, ecophysiology, genome‐wide association studies (GWAS), *Medicago truncatula*, plant microbiome, plant nutritional strategy, random forest, rhizosphere

## Abstract

- Harnessing the plant microbiome through plant genetics is of increasing interest to those seeking to improve plant nutrition and health. While genome‐wide association studies (GWAS) have been conducted to identify plant genes driving the plant microbiome, more multidisciplinary studies are required to assess the relationships among plant genetics, plant microbiome and plant fitness.
- Using a metabarcoding approach, we characterized the rhizosphere bacterial communities of a core collection of 155 *Medicago truncatula* genotypes along with the plant phenotype and investigated the plant genetic effects through GWAS.
- The different genotypes within the *M. truncatula* core collection showed contrasting growth and nutritional strategies but few loci were associated with these ecophysiological traits. To go further, we described its associated rhizosphere bacterial communities, dominated by *Proteobacteria*, *Actinobacteria* and *Bacteroidetes*, and defined a core rhizosphere bacterial community. Next, the occurrences of bacterial candidates predicting plant ecophysiological traits of interest were identified using random forest analyses. Some of them were heritable and plant loci were identified, pinpointing genes related to response to hormone stimulus, systemic acquired resistance, response to stress, nutrient starvation or transport, and root development.
- Together, these results suggest that plant genetics can affect plant growth and nutritional strategies by harnessing keystone bacteria in a well‐connected interaction network.

## Introduction

While conventional intensive agricultural practices allowed yields to drastically increase to feed a growing population, this relied mainly on plant breeding and a large use of inputs (e.g. fertilizers and pesticides). However, inputs have negatively impacted the environment, biodiversity and human health. Now agricultural production faces the challenge of supplying an increasing world population without hampering ecosystems (Tilman *et al*., 2002). Within the agroecological transition, one objective is to reduce the use of inputs and increase biological diversity and biological regulation without hampering crop production, the nature‐based solution (FAO, 2014).

In this context, there is a growing interest in maximizing ecosystemic services through the promotion of beneficial biological interactions, such as plant–microbiome interactions (Singh *et al*., 2020). The plant microbiome has been shown to improve plant nutrition and health (Lugtenberg & Kamilova, 2009; Bulgarelli *et al*., 2013; Pieterse *et al*., 2014). However, plant microbiome is driven by soil type, agricultural practices, environmental conditions, and biotic interactions (Philippot *et al*., 2013). Plant–microbiome interactions are thereby complex as a plant can also drive its associated microbiome, especially through plant genetics and molecular mechanisms. For example, it has been shown that plant species, plant genotype (Van Overbeek & Van Elsas, 2008; Micallef *et al*., 2009), plant compartment (Brown *et al*., 2020; Trivedi *et al*., 2020), plant developmental stage (Mougel *et al*., 2006; Edwards *et al*., 2018) and root exudates (Zhalnina *et al*., 2018) affect the plant microbiome. Therefore, increasing our knowledge is needed to identify the genetic bases and molecular mechanisms involved in the microbiome recruitment to be able to harness plant microbiome through plant genetics.

For that, two types of approaches have been developed (Bergelson *et al*., 2021): targeted approaches using mutants and transgenic lines in specific functions; and untargeted approaches using segregating or natural population and quantitative genetics (Horton *et al*., 2014; Escudero‐Martinez *et al*., 2022). Thereby, genome‐wide association studies (GWAS) have been used to identify new loci and genes impacting the microbiome (especially bacterial and fungal communities) in different model or cultivated plant species (i.e. *Arabidospis thaliana*, maize, rice, sorghum, foxtail millet and switchgrass) and for different plant compartments, such as leaves, roots and more recently the rhizosphere (Horton *et al*., 2014; Wallace *et al*., 2018; Bergelson *et al*., 2019; Roman‐Reyna *et al*., 2020; Deng *et al*., 2021; Brachi *et al*., 2022; Meier *et al*., 2022; Sutherland *et al*., 2022; VanWallendael *et al*., 2022; Wang *et al*., 2022; Andreo‐Jimenez *et al*., 2023; Su *et al*., 2024). These studies showed heritability especially for beta‐diversity components, specific OTUs/ASVs (operational taxonomic unit/amplicon sequence variant) or functions, but rarely on alpha‐diversity metrics. Plant genetic bases of microbial interactions were also recently assessed using microbial networks (He *et al*., 2021; Li *et al*., 2022). These studies enabled the identification of plant genes related to defense response, kinase activity, cell‐wall integrity, root development, trichome formation and nutrition.

While GWAS allowed identifying genes involved in microbiome recruitment to improve plant growth, nutrition, and health, interestingly few recent studies have assessed the effect of plant genetics on the associated microbiome in relation to plant performance (i.e. for *Arabidopsis* leaf bacterial and fungal communities on mature stem size by image analysis, a proxy for seed production, Brachi *et al*., 2022; for maize rhizosphere bacterial communities on 15 plant vigor traits, Meier *et al*., 2022; for foxtail millet root bacterial communities on 12 plant vigor traits, Wang *et al*., 2022; and for switchgrass rhizosphere bacterial communities on anthesis date and plant height, Sutherland *et al*., 2022).

In this study, we conducted the first GWAS analysis on the microbiome associated to the model legume, *Medicago truncatula*, and also considering both plant growth and plant nutritional strategy. We previously showed that the genotype of *M. truncatula* is affecting especially the rhizosphere bacterial communities, when analyzing both bacterial and fungal communities in the rhizosphere and in the root compartments (i.e. pooling rhizoplane and endosphere) (Zancarini *et al*., 2013). Therefore, we decided to conduct these GWAS analyses only on the rhizosphere bacterial communities using 16S rRNA gene sequencing and using a core collection of 155 accessions of *M. truncatula* grown in a Mediterranean soil under controlled glasshouse conditions. First, we characterized the different genotypes of the *M. truncatula* core collection for their growth and nutritional strategies, and identified their associated plant genetic loci using GWAS. Second, we described their associated rhizosphere bacterial communities, which can be considered as the ‘extended plant phenotype’. Then, we assessed relationships between the plant ecophysiological traits and their associated rhizosphere bacterial community composition to identify bacterial candidates predicting plant phenotypic traits of interest. Finally, we tested whether plant genetic loci are associated with these individual bacterial candidates through GWAS. Our study linked plant single nucleotide polymorphisms (SNPs), its associated rhizosphere bacterial community and plant growth and nutritional strategy.

## Materials and Methods

### Plant material and culture conditions

Seeds of 155 accessions of a *Medicago truncatula* core collection (Supporting Information Table S1) were scarified and surface sterilized (Mougel *et al*., 2006), vernalized at 4°C during 48 h, germinated on 0.7% (w/v) water agar plates at 25°C in the dark, and sown in a Mediterranean silty‐clayey soil (Mas d'Imbert, France) in a randomized design (Table S2). The plants were watered with demineralized water and cultivated up to the end of their vegetative period under a photoperiod of 14 h and a temperature of 25°C : 19°C (day : night).

### Plant phenotyping

At three sampling dates during the vegetative developmental stage (488, 734 and 848 degree‐days, or, respectively, 28, 42 and 49 d after sowing), three plants per accession were harvested to measure dry shoot and root biomasses, degree of nodulation (Moreau *et al*., 2008) and total carbon and nitrogen contents using a CHN (Carbon Hydrogen Nitrogen) analyzer (Carlo Erba, Val de Reuil, France). Then, the nitrogen nutrition index (NNI) was calculated for the last vegetative stage (Gastal & Lemaire, 2002; Moreau *et al*., 2008), and four parameters were estimated using an ecophysiological framework (i.e. the radiation use efficiency (RUE) for biomass production, the root to total biomass ratio (RTR), the plant‐specific N uptake (SNU), and the conversion factor of N to leaf area (NLA), Moreau *et al*., 2012). A different plant batch of three plants per accession was used to estimate leaf area every week from 19 to 54 d after sowing using a noninvasive monitoring setup (Moreau *et al*., 2009).

### Bacterial diversity, composition, functional prediction, and potential interactions

At the last harvest, plants were also analyzed to assess their associated rhizosphere bacterial community composition and diversity using an amplicon‐sequencing approach. DNA was extracted and quantified from rhizosphere soil as previously described by Mougel *et al*. (2006). The variable region V4 of the 16S rRNA gene was amplified using the F479 and R888 primers (Terrat *et al*., 2015) and sequenced using Illumina MiSeq sequencing technology by GenoScreen (Lille, France, https://www.genoscreen.fr/fr/). Then, the bioinformatic analyses were performed using the GnS‐PIPE, now renamed BIOCOM‐PIPE (Terrat *et al*., 2012; Djemiel *et al*., 2020). Next, the OTUs with counts lower than 41 over all the samples were filtered out, and a total count between‐sample normalization was applied to correct for the different sequencing depth.

Alpha‐diversity indices were calculated using the package vegan (Oksanen *et al*., 2020). While we used the nonfiltered and non‐normalized OTU occurrence dataset for the rarefaction curves, the observed richness and the Chao1 (Deng *et al*., 2024), we used the filtered and normalized OTU occurrence dataset for the Shannon, Pielou's evenness and inverse Simpson indices (Fig. S1).

The beta‐diversity was estimated at the OTU level based on both Bray‐Curtis and Sørensen distances (Baselga, 2010) using vegan and betapart packages (Baselga & Orme, 2012), respectively.

Functional prediction of the bacterial communities was assessed using PICRUSt2 (Phylogenetic Investigation of Communities by Reconstruction of Unobserved States) (Langille *et al*., 2013; Douglas *et al*., 2020). Correspondence between EC and Kyoto Encyclopedia of Genes and Genomes (KEGG) pathway and categories were downloaded from https://www.genome.jp/kegg‐bin/get_htext#B3 (version 14 October 2020) in order to be able to group EC per KEGG categories.

Finally, we calculated Spearman correlations using the SparCC method (Sparse Correlations for Compositional data (Friedman & Alm, 2012), using 500 iterations and 500 bootstraps) among the filtered and normalized occurrences of the rhizosphere bacterial OTUs, which are present in all the samples (*n* = 435), to define the core bacterial community of *M. truncatula*. Only significant correlations (pseudo *P*‐values ≤ 0.05 based on 500 bootstraps) with an absolute correlation magnitude ≥ 0.5 were considered for the network display using cytoscape software (Shannon *et al*., 2003). Network properties (e.g. density, diameter, mean distance, transitivity, assortativity, betweenness, eigen‐centrality and cliques) and the 1000 random networks were calculated using the igraph package (Csardi & Nepusz, 2006). Groups of OTUs were defined using the betweenness edge clustering method and hubs were pinpointed based on both their degree and betweenness centrality.

### Random forests

We conducted three regression random forests (RF) (Breiman, 2001) analyses using the muvr package (Shi *et al*., 2019) to identify the major OTU predictors for three plant phenotypic variables (total dry biomass at 848 degree‐days after sowing (TDW VS3), RTR and SNU). To decrease the number of OTU used in the RF analyses, we removed rare OTUs (< 0.01%) (Fig. S1). The statistical significance of the models was assessed with 100 permutations of the plant phenotypic variable.

### Estimates of genetic variance and heritability

Heritability (*h* ^2^) was calculated as genetic variance (var_G_) divided by the sum of genetic variance and the error variance (var_G_ + var_error_/*n*), where *n* = 3 is the total number of replicates in our random design. We considered as heritable a variable with significant nonzero genetic variance (*P*‐value of the likelihood ratio test for the genetic effect lower than 0.05) and *h* ^2^ higher than 0.25. Furthermore, we checked the variation within the replicates by calculating their coefficients of variation, and removed traits for which the median of their coefficients of variation over the different plant genotypes was greater than 0.6.

### Procedure for GWAS

SNP data were obtained by Illumina sequencing from the *Medicago truncatula* HapMap project (Stanton‐Geddes *et al*., 2013) and were filtered and imputed as described by Le Signor *et al*. (2017). The Gemma (Genome‐wide Efficient Mixed Model Association algorithm) software v.0.94 (Zhou & Stephens, 2012) was used to test through a standard linear mixed model for marker association with a single phenotype accounting for sample structure (Bonhomme *et al*., 2014) and population stratification. The positions of SNPs inside or in the vicinity of genes were downloaded from https://medicago.legumeinfo.org/. To refine candidate gene selection, a GEMMA *P*‐value cut‐off at 10^−6^ was applied to reduce the false‐positive rate while retaining minor effects SNPs. Singular enrichment analyses (SEA) using an exact Fisher test were done with the topgo r package (Alexa & Rahnenfuhrer, 2016) using GO term annotations from *Medicago truncatula* v.4.0v1 (https://jgi.doe.gov, (Tang *et al*., 2014)).

Full details of materials and methods are provided in Methods S1. All datasets used to produce the analyses and figures and R scripts are available in Notes S1. A list of the different acronyms used is provided in Table S3.

## Results

### Plant growth and nutritional strategies are contrasted within the *Medicago truncatula* core collection

**Fig. 1:**
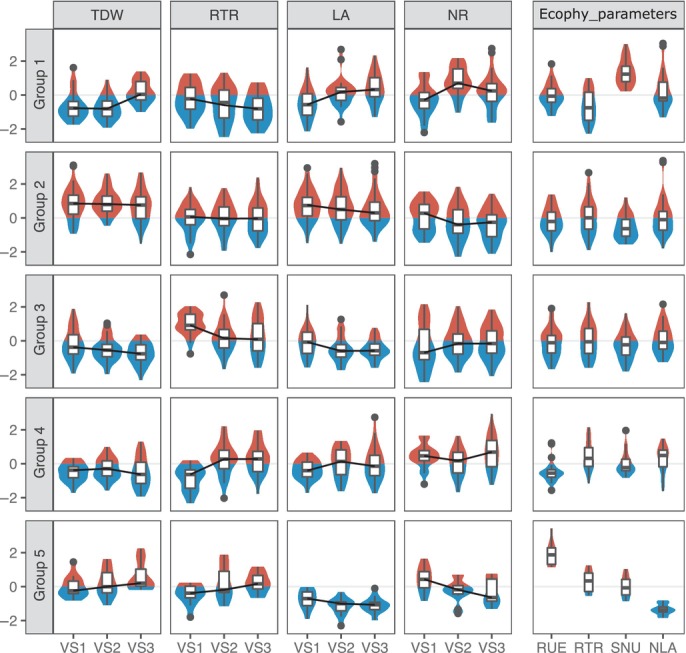
Plant growth and nutritional strategies of the five groups of plant genotypes. Five groups of plant genotypes were defined using the hclust ‘ward.D2’ linkage method in R software based on the ecophysiological dataset including only the non highly correlated variables (i.e. LA VS1, TDW VS1, RTR VS1, NR VS1, TDW VS2, RTR VS2, NR VS2, LA VS3, TDW VS3, NNI VS3, RUE, RTR, and SNU). Violin and boxplots were plotted using the average data per plant genotype (*n* = 146) that have been centered and scaled for each ecophysiological variable. The red and blue parts of the violin plots represent the portion of the violin plot with positive and negative values, respectively. The black line represents the median value for the 146 plant genotypes. The lower and upper hinges correspond to the first and third quartiles. The upper and lower whiskers extend from the hinge to the largest and smallest value, respectively (no further than 1.5 times the inter‐quartile range). Data beyond the end of the whiskers are plotted individually as black dots. Ecophy_parameters, ecophysiological parameters calculated; LA, leaf area (cm^2^); NLA, conversion factor of nitrogen to leaf area (cm^2^ of leaf g^−1^ of N); NR, nitrogen ratio (%); RTR, root to total biomass ratio; RUE, radiation use efficiency (g of total dry biomass MJ^−1^ of intercepted PAR); SNU, plant‐specific nitrogen uptake (g N g^−1^ of belowground dry biomass d^−1^); TDW, total dry weight (g); VS1, vegetative developmental stage 1 (488 degree‐days or 28 d after sowing); VS2, vegetative developmental stage 2 (734 degree‐days or 42 d after sowing); VS3, vegetative developmental stage 3 (848 degree‐days or 49 d after sowing).

Plant growth was characterized with standard phenotypic structural descriptors such as leaf area, shoot, root and total dry weight at three vegetative stages. *Medicago truncatula* showed significant contrasted biomasses and growth according to its genotype (Kruskal–Wallis tests, *P*‐value < 0.001, Table 1). Moreover, under our experimental conditions, all plant genotypes of the core collection exhibited N deficiency (nitrogen nutritional index (NNI) = 0.58 ± 0.03) (Table 1), while they all showed functional nodulated roots (i.e. many pink nodules of large size). Whereas no significant difference could be assessed in the observed nodulation score at the last vegetative stage, significant differences in nodulation scores at the two first vegetative stages were recorded among the plant genotypes (Kruskal–Wallis test, *P*‐value = 0.01 and 0.007, respectively). A significant plant genotype effect was also observed for NNI at VS3 (Kruskal–Wallis test, *P*‐value = 0.01). Finally, to go further and assess carbon (C) and nitrogen (N) nutritional strategies, an ecophysiological framework (Moreau *et al*., 2012) was used to estimate four parameters: RUE; RTR; SNU and the NLA (Table 1). Following a clustering analysis, based on Euclidean distances, five groups of plant genotypes showing contrasted growth and nutritional strategies were defined that did not correspond to the genetic structure of the core collection studied (Table S4). Group 2 is characterized by a high biomass along the three vegetative stages and a high‐nodulation score at the first vegetative stage (Fig. 1; Table 1). Groups 1 and 5 had low and medium biomasses at the first vegetative stage, but high biomasses at the last vegetative stage. Group 1 is characterized by a high SNU and low RTR, while group 5 is characterized by high RUE and RTR, and low NLA. Groups 3 and 4 are characterized by low biomasses. Group 3 showed low‐nitrogen ratio and nodulation score at the first two vegetative stages, and group 4 displayed both high RTR and low RUE (Fig. 1; Table 1).

**Table 1:** Plant growth and nutritional strategies of the *Medicago truncatula* core collection.

		Average ± SD	Kruskal–Wallis tests Pr (> *F*)		Average and Dunn tests				
			Plant genotype	Ecophy. group	Group E1	Group E2	Group E3	Group E4	Group E5
Leaf area (cm^2^)	VS1	5.7 ± 2.2	4 × 10^−4^ ***	5 × 10^−9^ ***	4.6 b	7.4 a	5.4 b	4.9 b	4.1 b
	VS2	28.6 ± 8.3	3 × 10^−6^ ***	3 × 10^−9^ ***	29.7 ab	33.2 a	24.2 bc	28.5 ab	18.9 c
	VS3	49.2 ± 14.5	9 × 10^−7^ ***	7 × 10^−9^ ***	54.0 a	55.0 a	41.5 bc	48.4 ab	33.6 c
Shoot dry biomass (g)	VS1	0.037 ± 0.016	3 × 10^−6^ ***	2 × 10^−10^ ***	0.027 b	0.051 a	0.031 b	0.032 b	0.038 ab
	VS2	0.25 ± 0.06	2 × 10^−5^ ***	3 × 10^−13^ ***	0.21 b	0.32 a	0.22 b	0.23 b	0.26 ab
	VS3	0.42 ± 0.10	3 × 10^−5^ ***	5 × 10^−11^ ***	0.44 a	0.47 a	0.34 b	0.36 b	0.46 a
Root dry biomass (g)	VS1	0.020 ± 0.009	4 × 10^−6^ ***	2 × 10^−10^ ***	0.014 b	0.026 a	0.021 a	0.013 b	0.019 ab
	VS2	0.11 ± 0.03	4 × 10^−5^ ***	9 × 10^−16^ ***	0.08 c	0.14 a	0.10 bc	0.11 b	0.12 ab
	VS3	0.20 ± 0.05	9 × 10^−4^ ***	1 × 10^−8^ ***	0.19 b	0.23 a	0.17 b	0.18 b	0.23 a
Total dry biomass (g)	VS1	0.057 ± 0.024	1 × 10^−6^ ***	3 × 10^−10^ ***	0.041 b	0.077 a	0.052 b	0.045 b	0.057 ab
	VS2	0.36 ± 0.09	1 × 10^−5^ ***	1 × 10^−14^ ***	0.30 c	0.45 a	0.32 bc	0.34 bc	0.38 ab
	VS3	0.62 ± 0.14	4 × 10^−5^ ***	3 × 10^−11^ ***	0.63 ab	0.70 a	0.52 c	0.55 bc	0.69 a
Root to total dry biomass ratio (RTR)	VS1	0.34 ± 0.05	0.02*	4 × 10^−13^ ***	0.33 bc	0.34 b	0.39 a	0.29 c	0.32 bc
	VS2	0.30 ± 0.03	0.32 ns	0.03*	0.28 b	0.30 ab	0.31 ab	0.31 a	0.31 ab
	VS3	0.33 ± 0.03	0.008**	9 × 10^−4^***	0.30 b	0.33 a	0.33 a	0.34 a	0.33 a
Nitrogen ratio (%)	VS1	3.8 ± 0.3	0.05 ns	0.004**	3.7 ab	3.8 ab	3.7 b	3.9 a	3.9 ab
	VS2	3.2 ± 0.2	0.02*	3 × 10^−4^***	3.4 a	3.1 b	3.2 b	3.2 ab	3.1 b
	VS3	3.2 ± 0.2	0.07 ns	0.003**	3.2 ab	3.1 b	3.1 ab	3.3 a	3.1 ab
NNI	VS3	0.58 ± 0.03	0.01*	0.01*	0.58 ab	0.57 b	0.58 ab	0.60 a	0.57 ab
Nodulation score	VS1	2.5 ± 0.7	0.01*	0.01*	2.3 ab	2.7 a	2.2 b	2.6 ab	2.8 ab
	VS2	3.7 ± 0.4	0.007**	0.009**	3.7 ab	3.8 ab	3.6 b	3.6 ab	3.9 a
	VS3	3.7 ± 0.3	0.36 ns	0.35 ns	3.7 a	3.7 a	3.6 a	3.7 a	3.8 a
RUE (g of total dry biomass MJ^−1^ of intercepted PAR)		2.9 ± 0.9	–	3 × 10^−7^***	2.9 b	2.8 b	2.8 b	2.5 b	4.7 a
RTR		0.32 ± 0.04	–	0.001**	0.30 b	0.32 ab	0.32 ab	0.34 a	0.33 a
SNU (mg of N g^−1^ of root biomass d^‐1^)		0.0089 ± 0.0023	–	2 × 10^−11^***	0.0115 a	0.0077 b	0.0081 b	0.0087 b	0.0087 b
NLA (cm^2^ of leaves g^−1^ of N)		2451 ± 714	–	6 × 10^−6^***	2661 a	2506 a	2525 a	2658 a	1542 b

Averages and SD were calculated for each variable using the mean values per plant genotype. Significance of Kruskal‐Wallis tests: ns, *, ** and *** indicate not significant and significant levels at 0.05, 0.01 and 0.001, respectively. Letters with different labels indicate significant differences (*P* < 0.05) for Dunn tests. Nodulation was visually assessed, using a qualitative scale (Moreau *et al*., 2008). Ecophy./E, ecophysiological; NLA, conversion factor of nitrogen to leaf area; NNI, nitrogen nutritional index; RUE, radiation use efficiency; SNU, specific nitrogen uptake; VS1, vegetative developmental stage 1 (488 degree‐days or 28 d after sowing); VS2, vegetative developmental stage 2 (734 degree‐days or 42 d after sowing); VS3, vegetative developmental stage 3 (848 degree‐days or 49 d after sowing).

### Plant loci associated with the plant ecophysiological traits

**Fig. 2:**
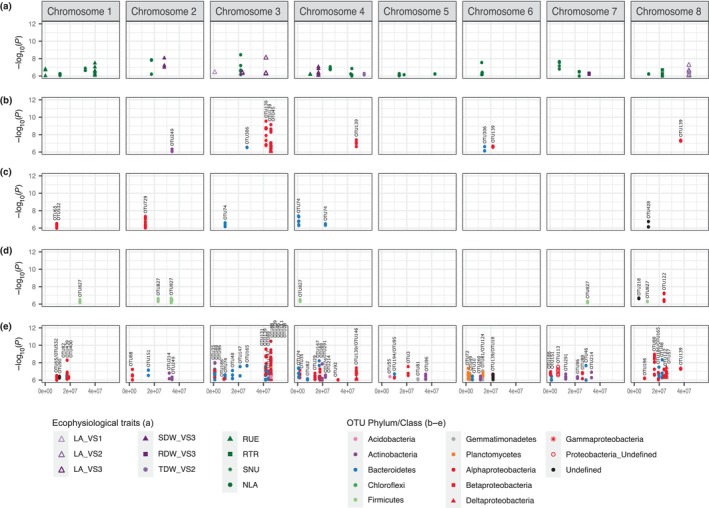
Loci associated to the ecophysiological variables and the rhizosphere bacterial operational taxonomic units (OTUs). (a) To reveal plant genes involved in plant growth and nutritional strategies, genome‐wide association studies (GWAS) were performed on all the different plant ecophysiological variables after square‐root transformation, and single nucleotide polymorphism (SNP)*phenotypic variable associations were selected based on the presence of at least 3 SNPs at a *P*‐value threshold of 10^−6^ within a 30 kB interval. To reveal plant genes associated with the OTUs predicting a plant ecophysiological trait, GWAS were performed on counts after square‐root transformation of the OTUs selected in the random forest predicting total biomass at VS3 (TDW VS3) (b), root to total biomass ratio (RTR) (c) and specific nitrogen uptake (SNU) (d). SNP*OTU associations were selected based on the presence of at least 3 SNPs at a *P*‐value threshold of 10^−6^ within a 30 kB interval. (e) To reveal plant genes involved in the composition of the bacterial community of *Medicago truncatula*, a GWAS was performed on the counts of the 64 heritable OTUs present in the co‐occurrence network and on the 71 heritable OTUs within the top one percent most abundant ones after square‐root transformation. SNP*OTU associations were selected based on the presence of at least 3 SNPs at a *P*‐value threshold of 10^−6^ within a 30 kB interval.

As plant biomass and nutritional strategies are shaped by the host genotype, we then performed GWAS on heritable traits (i.e. significant nonzero genetic variance and heritability higher than 0.25, Table S5) or parameters calculated from heritable variables (i.e. RUE, RTR, SNU, NLA) and for which the median of their coefficient of variation for the different plant genotypes was lower than 0.6 (Fig. S2) to identify genetic loci underlying variation in the plant phenotype using the plant ecophysiological variables, after square‐root transformation. A total of 665 SNP*phenotypic variable associations, representing 14 ecophysiological variables and 285 genes, were significant at *P*‐value threshold of 10^−6^. The 10^−6^ cut‐off was previously calculated by Bonhomme *et al*. (2014) based on the number of linkage disequilibrium blocks in the *M. truncatula* genome. To further refine SNP selection, hotspots of SNPs were selected for each ecophysiological variable based on the presence of at least 3 SNPs at *P*‐value thresholds of 10^−6^ within a 30 kB interval. Thereby, 161 hotspot SNP*phenotypic variable associations, representing 10 phenotypic variables and 50 genes were still significant after the filtering step (Fig. 2a). For leaf area at VS2, one hotspot was pinpointed on chromosome 8, underlying four genes, including one involved in multidimensional cell growth (Medtr8g104010). For RUE, three hotspots were pinpointed on chromosome 1 and 4, underlying seven genes, including one involved in response to light stimulus (Medtr1g088885) and two in defense response (Medtr1g007110 and Medtr4g029110). For NLA, one hotspot was pinpointed on chromosome 8 underlying one gene, Medtr8g031000, described as a bestrophin‐like protein. For RTR, one hotspot was pinpointed on chromosome 8 underlying three genes, including one involved in defense response, cell death, leaf senescence and ethylene biosynthetic process (Medtr8g463890). For SNU, several hotspots were pinpointed on all the chromosomes except chromosome 8, underlying 21 genes, including one involved in root hair cell development (Medtr4g069390), two in nodulation (Medtr7g063850 and Medtr7g063880) and one involved in defense response and transition from vegetative to reproductive phase (Medtr3g055370) (Tables S6, S7).

### Rhizosphere bacterial communities differ within the *M. truncatula* core collection, but a core rhizosphere bacterial community could be identified

In a previous study, we have shown that the genotype of *M. truncatula* particularly affects the rhizosphere bacterial communities, when analyzing both bacterial and fungal communities in the rhizosphere and in the root compartment for seven genotypes of *M. truncatula* (Zancarini *et al*., 2013). Therefore, in this study, we described the rhizosphere bacterial communities associated with the core collection of *M. truncatula* to go further in the description of the plant phenotype and its genetic determinism.

#### Diversity and global structure and composition

**Fig. 3:**
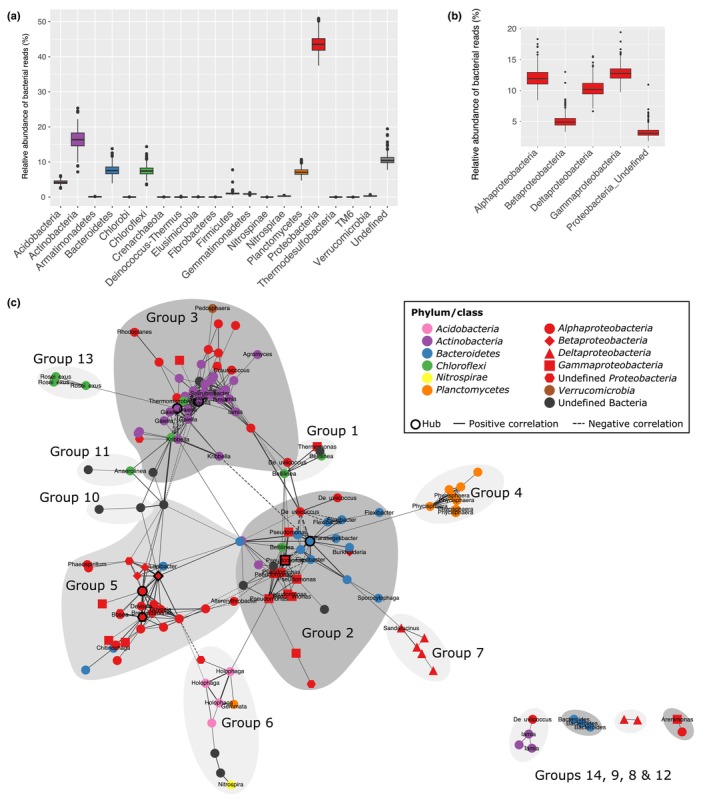
Description of the rhizosphere bacterial communities associated to the core collection of *Medicago truncatula*. (a) Composition of the rhizosphere bacterial communities of *M. truncatula*. Boxplots show the rhizosphere bacterial communities' occurrences at the phylum level associated with the *M. truncatula* core collection (*n* = 435). All operational taxonomic units (OTUs) belonging to the same phylum were summed for each sample using the normalized occurrence table. The black line represents the median value. The lower and upper hinges correspond to the first and third quartiles. The upper and lower whiskers extend from the hinge to the largest and smallest value, respectively (no further than 1.5 time the inter‐quartile range). Data beyond the end of the whiskers are plotted individually as black dots. (b) Composition of the rhizosphere *Proteobacterial* communities of *M. truncatula*. Boxplots show the rhizosphere *Proteobacterial* communities' occurrences at the class level associated with the *M. truncatula* core collection (*n* = 435). All the OTUs belonging to same class within the *Proteobacteria* were summed for each sample using the normalized occurrence table. The black lines represent the median value. The lower and upper hinges correspond to the first and third quartiles. The upper and lower whiskers extend from the hinge to the largest and smallest value, respectively (no further than 1.5 times the inter‐quartile range). Data beyond the end of the whiskers are plotted individually as black dots. (c) Co‐occurrence network of the *M. truncatula* core rhizosphere bacterial community. Spearman correlations were calculated between core bacterial OTUs using the SparCC method. Only significant correlations (pseudo *P*‐values ≤ 0.05 based on 500 bootstraps) with an absolute correlation magnitude ≥ 0.5 were considered for the network display using cytoscape. The network was visualized using the edge‐weighted spring embedded layout algorithm in cytoscape without forcing by correlation weight values. Nodes represent bacterial OTUs. The genus is written on each node (if known). Line thickness is proportional to the value of correlations between two nodes; thick and thin lines correspond to high (close to |1|) and low (close to |0.5|) correlations, respectively. The 14 groups of OTUs have been defined using the betweenness edge method.

First, after the removal of low‐quality reads and chimeras on raw data, high observed richness was recorded for the rhizosphere bacterial communities associated with *M. truncatula* in the Mediterranean soil used for this experiment (7656 ± 632; Table S8). Then, after removing less‐abundant sequences and normalization, 48395.77 sequences per sample were used for the following analyses. The bacterial communities were dominated by *Proteobacteria* (on average 44% ± 2), followed by *Actinobacteria* (on average 16% ± 3), *Bacteroidetes* (on average 8% ± 2), *Chloroflexi* (on average 8% ± 1), and *Planctomycetes* (on average 7% ± 1) (Fig. 3a). Among the *Proteobacteria*, the classes of *Gammaproteobacteria*, *Alphaproteobacteria* and *Deltaproteobacteria* contained on average 13% ± 1, 12% ± 1 and 10% ± 1 of the total sequences, respectively (Fig. 3b). *Rhizobiaceae* represented 0.01% of the total sequences (Table S9). Furthermore, our analyses revealed a beta‐diversity of 0.25 and a high turnover due to rare species (see Bray‐Curtis' dissimilarities, Whittaker's and Sørensen's indices in Table S8).

#### Potential functional prediction

Using PICRUSt2, we were able to predict functions that were potentially present in the rhizosphere bacterial communities of *M. truncatula*. After rarefying, we identified 2347 EC related to 228 KEGG pathways and 26 KEGG modules. Among them, amino acid metabolism, carbohydrate metabolism, metabolism of cofactors and vitamins, and the bride hierarchies' categories protein families: metabolism, genetic information processing, and signaling and cellular processes were the top KEGG functional categories identified (Fig. S3).

#### Core rhizosphere bacterial community

Looking at the bacterial OTUs present in all the samples, a core rhizosphere bacterial community was defined here for *M. truncatula*. To assess correlations within the core rhizosphere bacterial community, a co‐occurrence network analysis was performed. Among the 482 bacterial OTUs belonging to the core rhizosphere bacterial community, which represented 3% of the total OTUs but 76% of the total sequences, 150 displayed 358 positive and 36 negative correlations, resulting in a complex network (Fig. 3c). Nodes belonging to the same phylum (or class for *Proteobacteria*) were preferentially associated together, and hubs identified mainly belonged to *Proteobacteria* and *Actinobacteria* (*Gaiella*). Fourteen groups of OTUs have been defined using the betweenness edge method (Table S10) and only few OTUs connected the different groups (Fig. 3c). Moreover, several cliques of large size (between 6 and 9 nodes) were identified within the groups 2, 3 and 5. Finally, according to its density of 0.035, the co‐occurrence network had a significantly high diameter, mean distance, transitivity and assortativity (12, 4.2, 0.45 and 0.43, respectively) compared to 1000 random networks with the same number of nodes and density (Fig. S4a–d).

#### Plant genotype effect

The plant genotype structured its associated rhizosphere bacterial community diversity, composition and predicted potential functions. While alpha‐diversity showed significant differences according to *M. truncatula* genotype only for the Shannon and evenness indices (Kruskal–Wallis test, *P*‐value = 0.008 and 0.001, respectively, Table S8), the effect of the plant genotype explained 40% of the total variation in the beta‐diversity at the OTU level (PERMANOVA test with 9999 permutations, *R* ^2^ = 0.40 and *P*‐value = 1.10^−4^, Table S11). Moreover, occurrence at phylum level (or class for *Proteobacteria*) showed significant differences among plant genotypes on *Acidobacteria*, *Actinobacteria*, *Alphaproteobacteria*, *Bacteroidetes*, *Chloroflexi*, *Fibrobacteres*, *Gemmatimonadetes*, *Nitrospirae* and *Planctomycetes* (Kruskal–Wallis tests, *P*‐value = 1 × 10^−4^, 5 × 10^−3^, 0.02, 1 × 10^−5^, 0.01, 0.02, 8.10^−3^, 2 × 10^−3^ and 7 × 10^−6^, respectively, Table S12). Finally, a significant plant genotype effect could also be observed on the following KEGG categories of the associated bacterial communities: energy metabolism, amino acid metabolism, metabolism of other amino acids, glycan biosynthesis and metabolism, xenobiotics biodegradation and metabolism, transcription, translation, folding, sorting and degradation, replication and repair, signal transduction, cell motility, cellular community prokaryotes, drug resistance antimicrobial; protein families: metabolism, genetic information processing and signaling and cellular processes, and unclassified metabolism (Kruskal‐Wallis tests, *P*‐value = 8 × 10^−3^, 3 × 10^−4^, 4 × 10^−3^, 4 × 10^−4^, 5 × 10^−3^, 2 × 10^−4^, 0.02, 6 × 10^−4^, 0.05, 1 × 10^−3^, 8 × 10^−3^, 0.01, 5 × 10^−3^, 3 × 10^−3^, 4 × 10^−3^, 7 × 10^−5^ and 0.02, respectively, Table S12).

### Variations in the rhizosphere bacterial community and in the plant ecophysiological traits are closely associated

To assess to what extent variations in the rhizosphere bacterial community composition and the plant growth and nutritional strategies are associated, we ran both: a multivariate analysis to estimate the percentage of variation in the bacterial community structure harvested at the last vegetative stage (VS3 or 848 degree‐days after sowing) explained by the plant ecophysiological traits along the three vegetative stages; and a machine learning approach to identify candidate bacteria that can predict different plant ecophysiological traits at VS3.

**Fig. 4:**
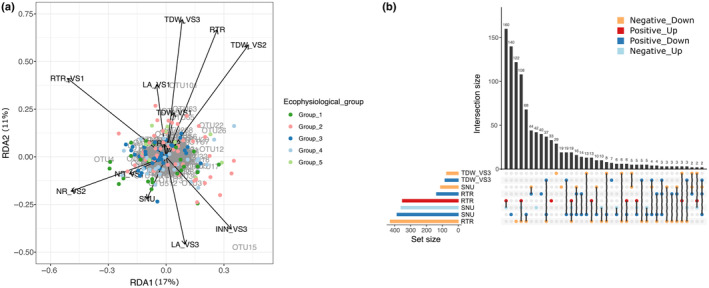
Relation between the rhizosphere bacterial communities and the plant phenotype. (a) Redundancy analysis and variation in plant growth and nutritional strategies that explain variation in the rhizosphere bacterial communities of *Medicago truncatula*. A redundancy analysis (RDA) was done using: (1) the filtered and normalized occurrence table summed per plant genotype (*n* = 155) as predicted variables; and (2) 13 of the plant phenotypic variables, which were not highly correlated (|Spearman correlation| < 0.6) as the explanatory variables. The plant phenotypic variables analyzed explained 10.3% of the total variation in the rhizosphere bacterial composition. Colored dots represent the 155 plant genotypes according to their corresponding ecophysiological group, arrows the ecophysiological variables and operational taxonomic units (OTUs) are in written in gray. LA, leaf area (cm^2^); NLA, conversion factor of nitrogen to leaf area (cm^2^ of leaf g^−1^ of N); NR, nitrogen ratio (%); RTR, root to total biomass ratio; RUE, radiation use efficiency (g of total dry biomass MJ^−1^ of intercepted PAR); SNU, plant‐specific nitrogen uptake (g N g^−1^ of belowground dry biomass day^−1^); TDW, total dry weight (g); VS1, vegetative developmental stage 1; VS2, vegetative developmental stage 2; VS3, vegetative developmental stage 3. (b) Number of potential enzyme classifications over/underrepresented in bacterial OTUs that are major predictors for plant ecophysiological variables. OTUs that are major predictors for the plant phenotype variables: TDW VS3, RTR and SNU, were first identified using a regression random forest (RF) analysis. In order to test if some functions were potentially more or less abundant for the candidate OTUs vs the rest of the bacterial communities, we considered the extended list of OTUs that are significantly correlated to the candidate OTUs identified by the random forests. This list of OTUs was then divided into two: OTUs positively and negatively correlated to the plant phenotype variable considered. A two‐sided *t*‐test was applied the EC_predicted.tsv output file from PICRUSt2 using the get*P*values function of topgo r package (Alexa & Rahnenfuhrer, 2016) to identify enzyme classifications (EC) that were potentially differentially abundant between the extended candidate OTUs list and the rest of the nonrare OTUs. The number of ECs is plotted using the upsetr package in r. Bars and dots colored: in dark red represent the number of EC more abundant in the extended candidate OTUs list than in the rest of the OTUs for the OTUs that are positively correlated to the plant phenotypic variable; in light red represent the number of EC less abundant in the extended candidate OTUs list than in the rest of the OTUs for the OTUs that are negatively correlated to the plant phenotypic variable; in dark blue represent the number of EC less abundant in the extended candidate OTUs list than in the rest of the OTUs for the OTUs that are positively correlated to the plant phenotypic variable; and in light blue represent the number of EC more abundant in the extended candidate OTUs list than in the rest of the OTUs for the OTUs that are negatively correlated to the plant phenotypic variable.

First and foremost, using a redundancy analysis (RDA), we showed that 10% of the variation in the bacterial community composition at VS3 could be explained by the variation in the plant phenotype (i.e. plant growth and nutritional strategy) (ANOVA‐like permutation test with 9999 permutations, *P*‐value = 0.0001, Table S13). Moreover, the nitrogen ratio at 734 degree‐days after sowing and RTR showed significant marginal effects in the RDA model (ANOVA‐like permutation test *P*‐value = 0.011 and 0.023, respectively; Table S13; Fig. 4a).

Then, using RF analyses, we pinpointed candidate bacterial OTUs that potentially predict plant phenotypic variables at VS3, such as total dry biomass (TDW VS3), RTR and SNU. These three variables were chosen to identify potential plant growth‐promoting rhizobacteria (predicting TDW and RTR) and bacteria that can improve nitrogen uptake via a biofertilization process (predicting SNU). Thereby, we identified 94 OTUs that were major predictors for plant biomass and nutritional strategies, within which 52 OTUs were identified as *Proteobacteria*, including 16 *Myxococcales* (*Deltaproteobacteria*) (Table S14). Next, from this narrow list, we defined an extended candidate list of 417 OTUs, including both the OTUs identified by the RF and their significantly correlated OTUs. Within this extended candidate list, bacterial OTUs identified as *Actinobacteria* (e.g. *Gaiellales*, *Solirubrobacterales* and undefined), *Bacteroidetes* (e.g. *Bacteroidales*, *Cytophagales* and *Sphingobacteriales*), *Alphaproteobacteria* (e.g. *Caulobacterales*, *Sphingomonadales*, *Rhodospirillales* and *Rhizobiales*), *Betaproteobacteria* (e.g. *Burkholderiales*) and *Deltaproteobacteria* (e.g. *Myxococcales*) were shown as positively or negatively associated with the different plant ecophysiological variables (Table S14). Finally, using an enrichment analysis, we identified potential functions (i.e. EC and KEGG pathways) that were more or less abundant for the candidate OTUs compared to the rest of the bacterial communities (Table S15). Potential functions differentially represented for the OTUs linked to RTR and SNU showed opposite results (Figs 4b, S5, S6). The extended candidate list of OTUs positively or negatively associated with RTR showed overrepresentation or underrepresentation, respectively, of potential functions involved in carbohydrate, energy, lipid, nucleotide and amino acid metabolisms, metabolism of other amino acids, metabolism of cofactor and vitamins (e.g. vitamin B6), metabolism of terpenoids and polyketides, biosynthesis of other secondary metabolism, xenobiotics biodegradation, transcription, replication and repair, signal transduction and cell motility (Figs S5, S6).

### Plant loci associated with rhizosphere bacterial OTUs

We previously showed that: ecophysiological traits (i.e. plant biomasses and nutritional strategies) and bacterial communities are shaped by host genotype; some plant loci were associated with the plant ecophysiological traits, especially for SNU; and that occurrence of some OTUs can predict some plant ecophysiological traits. To go further, GWAS were carried out on the occurrences of the heritable candidate OTUs (i.e. OTUs with significant nonzero genetic variance and heritability higher than 0.25, Table S16) with low variation among the three replicates (i.e. median of their coefficient of variation for the different plant genotypes lower than 0.6 (Fig. S7)) predicting the plant ecophysiological traits. We also ran GWAS on occurrences of the heritable OTUs of the core rhizosphere bacterial community network, the top 1% most abundant OTUs (157 OTUs) and on principal components (PC) of the community ordination plot to identify genetic loci underlying variation in the bacterial community.

#### GWAS analyses on the OTUs predicting the plant ecophysiological traits

To reveal genes involved in occurrence of the bacterial OTUs predicting total biomass (TDW VS3), RTR and SNU, GWAS was conducted on heritable OTU counts after square‐root transformation. A total of 482 SNP*OTU associations, representing 237 genes and 23 OTUs, were significant at a *P*‐value threshold of 10^−6^. To further refine SNPs selection, SNPs were selected for each OTU based on the presence of at least 3 SNPs at *P*‐value thresholds of 10^−6^ within a 30 kB interval. For the total biomass (TDW VS3), 8 hotspots on the chromosomes 2, 3, 4, 6 and 8 were pinpointed (Fig. 2b; Table S17), representing 84 SNP*OTU associations for 4 OTUs (OTU45/*Deltaproteobacteria*/*Myxococcales*, OTU139/*Gammaproteobacteria*/*Arenimonas*, OTU249/*Actinobacteria* and OTU306/*Bacteroidetes*/*Bacteroides*) and underlying 28 genes. Within the 20 annotated genes, four were involved in response to abscisic acid (Medtr3g092150, Medtr3g099920, Medtr3g100160 and Medtr3g100180), one was involved in triterpenoid biosynthetic process (Medtr3g092095), two in defense (Medtr3g092100 and Medtr3g092110), one was involved in root development (Medtr2g081520) and one in response to nitrate (Medtr8g089560) (Table S18). For the RTR, 50 SNP*OTU associations were pinpointed, representing 5 OTUs (OTU65/*Alphaproteobacteria*/*Sphingomonadaceae*, OTU74/*Bacteroidetes*/*Flexibacter*, OTU428/Undefined, OTU532/*Alphaproteobacteria*/*Sphingomonadales* and OTU729/*Alphaproteobacteria*/*Sphingomonadales*) and 11 genes, displayed on 6 hotspots on the chromosomes 1, 2, 3, 4 and 8 (Fig. 2c; Table S17). Within the 8 annotated genes, three were involved in response to abiotic or biotic stresses (Medtr1g027660, Medtr2g033730 and Medtr4g007030) (Table S18). For the SNU, 86 SNP*OTU associations, displaying 8 hotspots on the chromosomes 1, 2, 4, 7 and 8 and representing 3 OTUs (OTU122/*Alphaproteobacteria*/*Phaeospirillum*, OTU218/Undefinied and OTU827/*Firmicutes*/*Asteroleplasma*) and 40 genes, were pinpointed (Fig. 2d; Table S17). Within the 24 annotated genes, two were involved in lignin biosynthetic process (Medtr2g079580 and Medtr4g009690), one in cellular amide metabolic process (Medtr8g028125), two were involved in defense especially through salicylic acid (SA) (Medtr2g079670 and Medtr7g079180), three in response to abiotic stress (Medtr7g079020, Medtr7g079180 and Medtr8g028125) and one in root hair elongation (Medtr2g081180) (Table S18).

#### GWAS analyses on the bacterial community

To identify host genes that contribute to the structure of the bacterial community of *M. truncatula*, we used PCA after Hellinger transformation. The ordination plot distinguished the plant genotypes only when considering the top 10 to 1% most heavily sequenced OTUs (Fig. S8a), which represented > 87% to 64% of the total reads, respectively (Fig. S8b). Thus, GWAS was conducted on the first two PC of the PCA including only the top 10% most heavily sequenced OTUs, after square‐root transformation. Four SNP*PC associations were significant at a *P*‐value threshold of 10^−6^ (Fig. S8c; Table S17), representing one gene, Medtr4g018910 (verticillium wilt disease resistance protein).

#### GWAS analyses on heritable OTUs belonging to the co‐occurrence network and on the most abundant OTU

To reveal genes involved in the occurrence of both OTUs within the bacterial co‐occurrence network and the top 1% most abundant 157 bacterial OTU counts, GWAS was performed on heritable ones (Table S16) with low variation among the three replicates (Fig. S7) after square‐root transformation. A total of 1562 SNP*OTU associations were significant at a *P*‐value threshold of 10^−6^. To further refine the SNP selection, 570 SNP*OTU associations were selected based on the presence of at least 3 SNPs with a *P*‐value thresholds of 10^−6^ within a 30 kB interval, representing 143 genes and 41 OTUs (Fig. 2e; Table S17). For OTUs present in the co‐occurrence network, ontology analysis was run on the 108 genes identified and pointed out 22 significant overrepresented classes at classic Fisher *P*‐values < 0.05, including triterpenoid biosynthesis and metabolic process, response to SA, and response to nutrient levels and nitrate (Table S19). For OTUs present in the top 1% most abundant OTUs, ontology analysis was run on the 120 genes identified and pointed out 47 significant overrepresented classes at classic Fisher *P*‐values < 0.05, including response to extracellular stimulus, cellular response to nutrient levels and starvation (especially iron), and response to SA (Table S19). Within the 70 and 77 annotated genes for OTUs present in the co‐occurrence network and in the top 1% most abundant, 7 and 8 kinases were identified, respectively, with homologous of *A. thaliana* WAK2, CRK8, CRK25, CRK26, PEPKR2, ARK3 and AKIN10, zero and two LRR receptor‐like kinases, and two and two other LRR family proteins (diseases resistance protein). Moreover, within the seven genes related to SA response, one was kinase and two were MYB or MYB‐like transcription factors. Moreover, hotspots of SNP associated to different OTUs can be identified: on the chromosome 1 associated to OTU65/*Alphaproteobacteria/Sphingomonadaceae* and OTU532/*Alphaproteobacteria/Sphingomonadales*, on the chromosome 3 associated to OTU35/*Gammaproteobacteria/Xanthomonadales* and OTU48/*Bacteroidetes*/*Flexibacter*, to OTU165/*Bacteroidetes*/*Bacteroides* and OTU306/*Bacteroidetes*/*Bacteroides*, and to OTU88/*Alphaproteobacteria/Rhodospirillales*, OTU139/*Gammaproteobacteria*/*Arenimonas*, OTU45/*Deltaproteobacteria*/*Myxococcales*, OTU28/*Acidobacteria*/*Holophaga* and OTU111/*Planctomycetes/Gemmata*, and on the chromosome 4 associated to OTU74/ *Bacteroidetes*/*Flexibacter* and OTU214/*Actinobacteria*. While OTU65, OTU74 and OTU532 are predicting RTR, and OTU45, OTU139 and OTU306 are predicting TDW VS3 based on the random forest results, all the others OTUs in the hotspots are correlated to at least one OTU predicting at least one of the three ecophysiological traits analyzed in the random forest.

## Discussion

To develop new plant varieties that select for beneficial microbes in an agroecological context, using low inputs while maintaining yields, a better understanding of the genetic determinisms that drive the plant microbiome recruitment is needed. To our knowledge, six untargeted approaches using GWAS have been conducted to identify plant loci or genes associated with variation in the root, rhizoplane or rhizosphere microbiome (Bergelson *et al*., 2019; Deng *et al*., 2021; Meier *et al*., 2022; Sutherland *et al*., 2022; Wang *et al*., 2022; Andreo‐Jimenez *et al*., 2023). While the common final goal of these GWAS is to harness the beneficial microbiome through plant genetics, interestingly, half of them considered plant performance traits. Our study is the first one to assess the impact of the host genetics on the rhizosphere bacterial communities through a GWAS for a legume plant, *Medicago truncatula*, and in relation to plant growth and C and N nutritional strategy. Hence, here, we provide new information about associations among plant genetics, rhizosphere bacterial communities and plant phenotype.

### *Medicago truncatula* showed genetic variation in plant growth and nutritional strategy, but few plant loci could be identified for these integrative ecophysiological traits

To phenotype the core collection, we used both structural phenotypic descriptors, such as biomasses, and functional phenotypic descriptors, such as RUE and SNU, to describe both establishment of the plant structures (i.e. leaf area and roots) and their efficiency to uptake C and N. Indeed, we previously showed that functional descriptors enable the discrimination between genotypes with similar biomasses but with different nutritional strategies (Zancarini *et al*., 2012, 2013). Within the core collection of *M. truncatula*, five different groups of plant genotypes were defined according to their growth and nutritional strategies (Fig. 1; Table 1). First, group 1 showed significantly high SNU and low RTR and RUE. According to the ‘functional equilibrium’ model, the dry biomass distribution between root and shoot can be regulated by equilibrium between root activity (water and nutrient absorption) and shoot activity (photosynthesis) (Brouwer, 1983; Farrar & Jones, 2000). Therefore, we observed that, contrary to plants in group 5, plants within group 1 did not invest biomass in their roots (significantly low RTR), which were more efficient than the other groups in taking up N (significantly high SNU), but in their shoots, which were less efficient in taking up C than the other groups (significantly low RUE). These two opposite strategies (groups 1 and 5) seemed to be efficient with time as their total biomasses went from the lowest/average ones at the first harvest to the highest ones at the last harvest. This result suggests that investing in an efficient function to either take up N or C might be costly at early developmental stages, but later yields under our experimental conditions, where no nutrient was added in the watering solution.

Under our experimental conditions, without nitrogen starter addition to compensate for *M. truncatula* small and low‐reserve seeds, nor any addition of nitrogen in the watering solution, N uptake of the different genotypes of *M. truncatula* was not able to match the plant N requirements whereas: plants were in the presence of a mixture of *Ensifer* strains naturally present in the Mas d'Imbert soil, corresponding mostly to *Ensifer medicae* species (Rangin *et al*., 2008) that are considered as more efficient than *Ensifer meliloti* (Moreau *et al*., 2008; Terpolilli *et al*., 2008; Larrainzar *et al*., 2014); and we observed active nodules. However, a difference was observed in the nodulation score at early developmental stages for the plant groups 2 and 3 that showed higher and lower total biomass, respectively, along the three vegetative stages. These results suggest that a faster nodulation establishment under our experimental conditions seems to provide an advantage in term of growth development. Furthermore, *M. truncatula* has previously been defined as an efficient root forager (Batstone *et al*., 2017), results that we also observed previously in the same Mediterranean soil (Zancarini *et al*., 2012). Therefore, we can expect that using a different soil with a different chemical composition and microbial communities could also affect nodulation establishment and plant N nutritional status.

To identify the genetic determinisms involved in the observed variation in plant growth and nutritional strategies, GWAS were performed. Except for SNU, only a few hotspots and plant loci were highlighted for the different plant ecophysiological traits. The identified genes were mainly involved in either plant growth or in defense response.

### The rhizosphere bacterial community is an important trait of the plant phenotype, affected by both the plant genotype and the ecophysiological traits and affecting the latter

To assess the ‘extended plant phenotype’, we described the rhizosphere bacterial community diversity and composition. High observed richness for the rhizosphere bacterial communities were recorded in our study and the OTUs identified were not dominated by *Ensifer* contrary to the results of Brown *et al*. (2020). In fact, with our metabarcoding approach, *Rhizobiaceae* represented only 0.01% of the total sequences within the rhizosphere. However, Brown *et al*. (2020) collected their soil from the base of *Medicago* plants, where *Ensifer* species are expected to be enriched, while we collected our soil in fallow land, in which a mixture of plant species/families grows naturally, including legume and nonlegume plants.

Regarding the bacterial composition, according to the different plant species and soil assessed *Proteobacteria*, *Acidobacteria*, *Actinobacteria*, *Bacteroidetes*, *Firmicutes* and/or *Planctomycetes* are usually dominated the rhizosphere bacterial community (Bulgarelli *et al*., 2012, 2013; Lundberg *et al*., 2012; Peiffer *et al*., 2013; Edwards *et al*., 2015; Deng *et al*., 2021). While the effect of the soil on the microbial composition is well known (Lundberg *et al*., 2012), in accordance with the results on legumes published in a recent meta‐analysis (Ling *et al*., 2022), *Proteobacteria*, *Actinobacteria* and *Bacteroidetes* were shown to dominate the community associated to the core collection of *M. truncatula* in our Mediterranean soil. These phyla have been previously classified as copiotroph and shown as more competitive in a nutrient‐enriched environment like rhizosphere (Fierer *et al*., 2007; Pérez‐Jaramillo *et al*., 2016). Moreover, our results suggested that enzyme classification (EC) involved in amino acid metabolism, carbohydrate metabolism, metabolism of cofactors and vitamins are the major functions predicted for the rhizosphere bacterial community of *M. truncatula*.

Within the rhizosphere of the *M. truncatula* plants, a core rhizosphere bacterial community was identified and potential interactions within the core OTUs were depicted through a co‐occurrences network analysis. Only one third of the core rhizosphere bacterial OTUs showed significant potential interactions or direct/indirect correlations due to their similar environmental condition preferences. However, the core rhizosphere bacterial community network is significantly highly interconnected among the different OTUs. Several groups of highly positively correlated OTUs, belonging especially to the same taxa, were then identified, suggesting that they shared similar niches as has been previously shown in different habitats, such as in soil and in the human gut (Barberán *et al*., 2012; Faust *et al*., 2012). In line with a recent meta‐analysis (Ling *et al*., 2022), the hubs identified in our study belonged to both the *Proteobacteria* and *Actinobacteria* phyla, and more especially for the later to the *Gaiella* genus, which was also recently identified as a module hub in bulk soil (Ling *et al*., 2022).

Furthermore, while Brown *et al*. (2020) showed that plant genotype affects the structure of the root but not the rhizosphere bacterial communities associated with three genotypes of *M. truncatula* grown in two different Mediterranean soils (i.e. *M. truncatula* native range) using amplicon sequencing, we previously showed that plant genotype affects the rhizosphere bacterial communities but not the root bacterial and the fungal communities for seven other genotypes of *M. truncatula* grown in our Mediterranean soil using DNA fingerprints (Zancarini *et al*., 2013). This plant genotype effect on the rhizosphere bacterial communities has been confirmed in this study for a core collection of 155 *M. truncatula* genotypes using amplicon sequencing.

Finally, in addition to the effect of the plant genotype on the rhizosphere bacterial communities, we also showed that they are closely linked to the plant ecophysiological traits. On the one hand, the variation in the bacterial composition at the last vegetative stage can be partially explained by the plant traits, such as the nitrogen ratio at the second vegetative stage and RTR (Fig. 4a), and, on the other hand, that plant traits (TDW VS3, RTR, and SNU), can be predicted based on the bacterial occurrence using machine learning. Among the 94 candidate OTUs identified, 16 of them belong to *Myxococcales* (*Deltaproteobacteria*) and are negatively correlated to the total biomass and the SNU, and positively to the RTR. *Myxococcales* are known as micropredactors in soil and as important players in soil C sequestration and mineralization, which can be affected by organic matter, C : N ratio, total N and soil pH, and produce versatile specialized metabolites, such as carotenoids (Lueders *et al*., 2006; Zhou *et al*., 2014, 2020; Li *et al*., 2017; Wang *et al*., 2020; Lv *et al*., 2022; Nwachukwu *et al*., 2022). Our study also pinpointed potential new plant growth promoting bacteria, which are positively linked to the total biomass and/or RTR, and potential new plant nitrogen uptake promoting bacteria (positively linked to SNU) (Table S14). The metabarcoding approach used in our study on a natural soil community could not enable us to confirm and identify the potential bacterial functions that could explain a better plant biomass, RTR and/or SNU. Only isolation and characterization of these specific rhizosphere bacterial strains would help us in confirming these hypotheses. Indeed, for several years now, there has been a growing interest in developing microbial culture collections and isolating strains from the field and controlled condition experiments. Furthermore, it will be important in future studies to discriminate between direct and indirect effects among the different drivers involved in plant–microbiome interactions and go beyond associations to causation that our current biostatistical approaches do not allow. Indeed, when looking at the enzyme classifications enriched for the candidate bacteria predicting a bigger root system (higher RTR), they potentially express a more active metabolism (e.g. carbohydrate, energy, lipid, nucleotide and amino acid metabolisms, etc.) hinting at an adaptation to a richer environment. This result suggests that more than the presence of the candidate bacteria predicting the plant trait, the latter (i.e. higher RTR, thus potentially higher root surface and root exudates) seems to drive the selection of candidate bacteria (i.e. potentially more copiotroph). Wagner (2021) recently discussed the importance of considering plant phenotype both as a predictor and a readout in genetic studies on plant–microbiome interactions because plant genetics is expected to harness the microbiome more through a change in its phenotype than through a direct genetic effect.

### Plant genetic potential to improve plant growth, nutrition and defense harnessing keystone rhizosphere bacteria

To study the plant genetic determinism in its associated rhizosphere bacterial communities' structure and composition, plant loci and genes were identified using GWAS on heritable individual OTU occurrences such as: the candidate OTUs predicting the plant traits; the OTUs present in the co‐occurrence network; the most abundant OTUs; and a bacterial community proxy (principal component axes of the ordination plot).

**Fig. 5:**
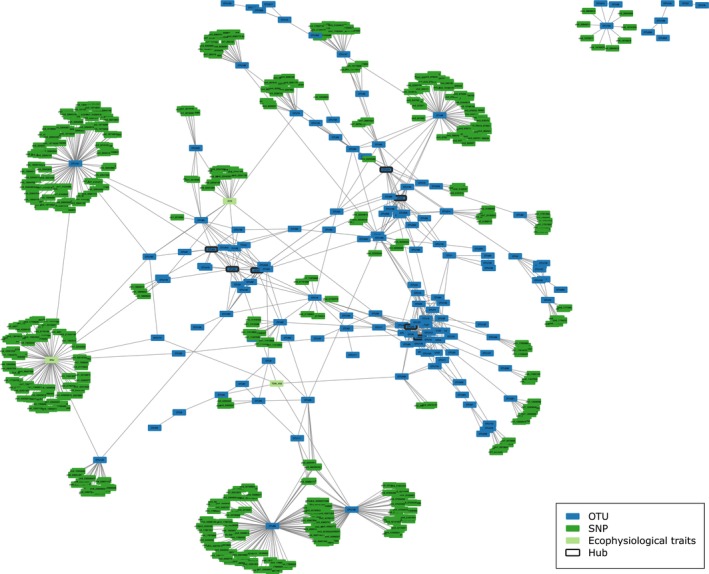
Associations among plant single nucleotide polymorphisms (SNPs), bacterial OTUs and ecophysiological variables. The network was visualized using cytoscape's edge‐weighted spring embedded layout algorithm without forcing by correlation weight values. Nodes represent bacterial OTUs(blue), plant SNPs (dark green) and ecophysiological traits (i.e. TDW, RTR and SNU in light green). OTU hubs are shown with a black highlighted border. OTU*OTU associations were the ones identified within the core bacterial OTUs using the SparCC method. Both SNP*OTU and SNP*ecophysiological variables associations identified by the genome‐wide association studies (GWAS) were selected based on the presence of at least 3 SNPs with a *P*‐value threshold of 10^−6^ within a 30 kB interval. Associations between the bacterial OTUs and the ecophysiological variables are those identified using the random forest analyses. RTR, root to total biomass ratio; SNU, specific nitrogen uptake (g N g^−1^ of belowground dry biomass d^−1^); TDW, total dry weight (g) at vegetative developmental stage 3 (848 degree‐days or 49 d after sowing).

First, our results suggested that there is a stronger plant genetic effect on individual bacteria than at the community level. Several authors discussed the importance of knowing if it would be possible to change the plant associated microbial community by driving only keystone taxa, or if a minimum level of community complexity is required (Agler *et al*., 2016; Van Der Heijden & Hartmann, 2016; Banerjee *et al*., 2018). In our study, within the 150 OTUs present in the co‐occurrence network, 30 OTUs were both heritable and significantly determined by the plant genotype. These results suggest that plants can genetically harness individual OTUs, which are correlated to other OTUs in a well‐connected network community. Moreover, these OTUs are also directly or indirectly linked to the plant ecophysiological traits (Fig. 5). Together, this highlights the plant genetic potential to improve plant growth and nutrition by harnessing keystone rhizosphere bacteria.

Then, we observed fewer plant loci associated with plant ecophysiological traits than with the occurrence of the individual bacteria. Moreover, no colocalization could be identified between the hotspots associated with the bacterial OTUs and the plant traits (Fig. 2). The genes identified were mainly related to hormone response (e.g. SA, abscisic acid), defense, triterpenoid biosynthesis and metabolic processes, response to nutrient level or transport (especially nitrate and iron), and root development.

More specifically, on the one hand, among the genes identified in our GWAS for bacterial OTUs, several of them were potentially involved in the establishment of the root microbiome and defense against pathogens. Indeed, nine of the genes identified through the GWAS, including two MYB‐like or MYB transcription factors with no clear gene to gene homology with *Arabidopsis thaliana*, were involved in response to SA, SA biosynthetic processes or SA mediated signaling pathway and were associated with the occurrence of OTUs belonging to *Acidobacteria*/*Holophaga*, *Bacteroidete*/*Chitinophagaceae*/*Parasegetibacter*, *Bacteroidete*/*Flexibacter*, *Firmicutes*/*Asteroleplasma*, *Alphaproteobacteria*/*Rhodospirales*, *Alphaproteobacteria*/*Sphingomonadales*, and *Gammaproteobacteria*/*Xanthomonadaceae*/*Arenimonas*. MYBs are known to play regulatory roles in developmental processes and defense responses in plants, and expression of half of MYB related genes identified in *A. thaliana* were responsive to SA treatment (Yanhui *et al*., 2006). Moreover, Lebeis *et al*. (2015) showed that among others *Rhodospirales*, *Sphingomonadales*, and *Xanthomonadaceae* are either enriched or depleted in SA *Arabidopsis* mutants. While SA pathways are required to assemble a normal root microbiome (Lebeis *et al*., 2015), they are also closely related to the systemic acquired resistance process (SAR), for which seven genes have been found in our study including five that are in common with SA processes. In addition, we found three genes involved in terpenoid transport and triterpenoid biosynthesis. Huang *et al*. (2019) showed that specialized triterpenes produced by *A. thaliana* can also affect its associated root bacterial community assembly and maintenance. Furthermore, regarding the defense process, 14 genes involved in defense mechanisms have been pinpointed by our GWAS analyses, including nucleotide‐binding‐leucine‐rich‐repeat (NB‐LRR) and NB‐ARC disease resistance protein, TIR‐NBS‐LRR protein and LRR receptor kinase protein. NB‐LRR receptor proteins are known as a second line of defense, the effector‐triggered immunity, mediated by pathogen effector recognition (Cui *et al*., 2015). Also, two genes belonging to the CRK (cysteine (Cys)‐rich receptor‐like kinases) family were found to be significantly associated with the OTU81 (*Gemmatimonas*) occurrence. In *Arabidopsis*, these membrane‐localized CRK proteins are synthesized upon pathogen perception and act coordinately to enhance plant immune responses (Yadeta *et al*., 2017).

On the other hand, regarding nutrition, four genes were related to response to nitrate and eight to iron associated with, among others, three *Xanthomonadaceae* in which Fe‐metabolizing bacteria are found. To assimilate iron, it has been shown for *A. thaliana* that plants can synthesize coumarins, which are known for their antimicrobial activities and roles in root‐microbiome communication, microbiome assembly and in nutrient uptake (Siwinska *et al*., 2018; Stringlis *et al*., 2018, 2019; Voges *et al*., 2019). Another gene associated with OTU74/*Flexibacter* has been pinpointed by our GWAS which is a proton‐pump interactor, putatively implicated in rhizosphere acidification, a mechanism necessary to increase iron solubility (Li *et al*., 2015). Finally, regarding growth, one gene associated with OTU139/*Arenimonas* is classified both as primary root development and nutrient detection, and one gene involved in glutathione (GSH) metabolism was found. Existence of an interplay between GSH and auxin in control of root growth has been shown as GSH depletion reduces root growth through inhibition of auxin transport (Koprivova *et al*., 2010). When there was IAA excess, GSH was important as a redox buffer and may reduce the concentration of reactive oxygen species that are necessary for root growth. Finally, four other genes were found and described as being involved in root hair elongation, differentiation, and root development. Together these results suggest a trade‐off among plant growth, nutrition and defense, and the importance of the different lines of defense in the establishment of the root‐associated microbiome.

In conclusion, we characterized a core collection of *M. truncatula* grown in a Mediterranean soil for their growth, nutritional strategies, and associated rhizosphere bacterial communities, traits that all varied significantly among the plant genotypes. At the plant genetic level, while a few plant loci were associated with the plant ecophysiological traits, several hotspots and loci were identified and associated with the occurrence of specific bacterial OTUs, which are correlated to other OTUs in a well‐connected network community. Furthermore, the occurrence of several of these bacterial OTUs was associated with plant ecophysiological traits of interest. Therefore, our results suggested that we can potentially drive both the bacterial community and the plant phenotype through a plant genetic determinism. The use of microbial collection and synthetic communities could help us to better understand the microbial functions driven by the plant, characterize the direct effect of the bacteria on the plant phenotype, and assess how these keystone bacteria affect the rest of the microbial community. Moreover, focusing on microbial functions more than microbial composition should allow us to find more universal results across different soils. Finally, to further understand plant genetic effects and the molecular mechanisms involved in beneficial microbiome recruitment, functional genomics is required to confirm our results and validate the plant genes identified in the recruitment of these keystone microbes and on the plant phenotype.

## Competing interests

None declared.

## Author contributions

AZ, CS, NM‐J and CM, participated to the conception and the design of the work; AZ performed experiments and organized data acquisition; AZ, CLS, ST and JA analyzed the data; AZ, CLS, CM, NM‐J and CS interpreted the results; AZ and CLS drafted the manuscript; AZ, CLS, ST, JA, CS and CM reviewed the manuscript; all the authors read and approved the final manuscript.

## Supporting information


**Fig. S1** Diagram representing the main analytical steps for both the plant ecophysiological and the bacterial communities' data and their genetic analyzes outputs.
**Fig. S2** Coefficients of variation for the ecophysiological traits.
**Fig. S3** Potential functions of the rhizosphere bacterial communities associated with the *Medicago truncatula* core collection.
**Fig. S4** Co‐occurrence network of the *Medicago truncatula* core rhizosphere bacterial community.
**Fig. S5** Potential KEGG pathways over/underrepresented in bacterial OTUs predicting *Medicago truncatula* ecophysiological traits.
**Fig. S6** Number of potential enzyme classification per KEGG modules over/underrepresented in bacterial OTU linked to *Medicago truncatula* phenotype.
**Fig. S7** Coefficients of variation for the heritable OTUs.
**Fig. S8** Genetic variation within *M. truncatula* shapes the composition of the most heavily sequenced OTUs of the rhizosphere bacterial communities.
**Methods S1** Detailed materials and methods.

**Notes S1** Zip file containing datasets and R scripts used to produce the analyses and figures.

**Table S1** Plant genotype list and their geographical origin.
**Table S2** Plant position in glasshouse.
**Table S3** Acronym list.
**Table S4** Plant phenotypic variables measured and calculated for each genotype of the core collection of *Medicago truncatula*.
**Table S5** Estimation of the heritability of the ecophysiological parameters.
**Table S6** GWAS output for the ecophysiological parameters.
**Table S7** List of Gene IDs corresponding to the significant SNPs in the GWAS for each ecophysiological traits and their corresponding annotation, GO id and GO term.
**Table S8** Richness, alpha‐ and beta‐diversity indices of the rhizosphere bacterial communities associated with the *Medicago truncatula* core collection.
**Table S9** Filtered and normalized occurrence table including all samples (*n* = 435) used in alpha‐ and beta‐diversity analyses, bacterial composition, and functional prediction.
**Table S10** Taxonomic affiliation, relative abundance, and properties in the co‐occurrence network of the 150 OTU found significantly correlated in the co‐occurrence network analysis.
**Table S11** PERMANOVA results based on Bray‐Curtis distances.
**Table S12** Plant genotype effect on bacterial composition at Phylum and Class levels and on KEGG categories.
**Table S13** Redundancy analysis on microbial data and ecophysiological for the core collection of *Medicago truncatula*.
**Table S14** List of the candidate OTU, which are found as major predictor of three plant phenotypic variables and their significantly correlated OTU.
**Table S15** Potential enzyme classification that are significantly more and less abundant for the OTU positively and negatively linked to the three plant phenotypic variables analyzed in random forest in comparison to the rest of the bacterial OTU and their associated KEGG categories and pathways.
**Table S16** Estimation of the heritability for the 900 most abundant OTUs.
**Table S17** Global GWAS output for the abundance of the bacterial OTUs (OTUs predicting plant ecophysiological traits, OTUs in the co‐occurrence network and the top 157 most abundant OTUs) and for the two first principal component axes of the ordination plot representing the bacterial community (using the top 10% more abundant OTU).
**Table S18** List of Gene IDs corresponding to the significant SNPs in the GWAS for each analysis of VIP OTU group predicting ecophysiological traits and their corresponding annotation, GO id and GO term.
**Table S19** List of Gene IDs corresponding to the significant SNPs in the GWAS for each OTU group (co‐occurrence network and top 157 most abundant ones), their corresponding annotation, GO id and GO term, and top GO output.
Please note: Wiley is not responsible for the content or functionality of any Supporting Information supplied by the authors. Any queries (other than missing material) should be directed to the *New Phytologist* Central Office.

## Data Availability

All raw metabarcoding datasets are publicly available in the European Nucleotide Archive (ENA) of European Molecular Biology Laboratory's European Bioinformatics Institute (EMBL‐EBI) database system under project accession no PRJEB25849. All the other datasets used to produce the analyses and figures and R scripts are available in Notes S1.
