# Supplementary material for: Medicago truncatula genotype drives the plant nutritional strategy and its associated rhizosphere bacterial communities
Source: New Phytol. 2024 Nov 28;245(2):767–84. doi: 10.1111/nph.20272 (PMC11655437; doi:10.1111/nph.20272)
Supplement: Supplementary file 1 — Fig. S1 Diagram representing the main analytical steps for both the plant ecophysiological and the bacterial communities' data and their genetic analyzes outputs. Fig. S2 Coefficients of variation for the ecophysiological traits. Fig. S3 Potential functions of the rhizosphere bacterial communities associated with the Medicago truncatula core collection. Fig. S4 Co‐occurrence network of the Medicago truncatula core rhizosphere bacterial community. Fig. S5 Potential KEGG pathways over/underrepresented in bacterial OTUs predicting Medicago truncatula ecophysiological traits. Fig. S6 Number of potential enzyme classification per KEGG modules over/underrepresented in bacterial OTU linked to Medicago truncatula phenotype. Fig. S7 Coefficients of variation for the heritable OTUs. Fig. S8 Genetic variation within M. truncatula shapes the composition of the most heavily sequenced OTUs of the rhizosphere bacterial communities. Methods S1 Detailed materials and methods. [file NPH-245-767-s001.pdf]

New Phytologist

***New Phytologist* Supporting Information**

Article title: *Medicago truncatula* genotype drives the plant nutritional strategy and its associated rhizosphere bacterial communities
Authors: Anouk ZANCARINI, Christine LE SIGNOR, Sébastien TERRAT, Julie AUBERT, Christophe SALON, Nathalie MUNIER-JOLAIN, and Christophe MOUGEL
Article acceptance date: 22 October 2024

The following Supporting Information is available for this article:

Methods S1 Detailed materials and Methods

Methods S1 Fig.1 Rarecurves

Methods S1 Fig. 2 QQplots for all the heritable OTU analyzed in GWAS

Methods S1 Table 1 Bioinformatic parameters and databases used in the metabarcoding analysis of 16S rRNA gene sequences

Methods S1 Table 2 Spearman correlations among the plant phenotypic variables measured and calculated for the core collection of *M. truncatula*

Fig. S1 Diagram representing the main analytical steps for both the plant ecophysiological and the bacterial communities' data and their genetic analyzes outputs

Fig. S2 Coefficients of variation for the ecophysiological traits

Fig. S3 Potential functions of the rhizosphere bacterial communities associated with the *Medicago truncatula* core collection

Fig. S4 Co-occurrence network of the *Medicago truncatula* core rhizosphere bacterial community

Fig. S5 Potential KEGG pathways over/under-represented in bacterial OTUs predicting *Medicago truncatula* ecophysiological traits

Fig. S6 Number of potential enzyme classification (EC) per KEGG modules over/under-represented in bacterial OTU linked to *Medicago truncatula* phenotype

Fig. S7 Coefficients of variation for the heritable OTUs

Fig. S8 Genetic variation within *M. truncatula* shapes the composition of the most heavily sequenced OTUs of the rhizosphere bacterial communities

Table S1 Plant genotype list and their geographical origin

Table S2 Plant position in glasshouse

Table S3 Acronym list

Table S4 Plant phenotypic variables measured and calculated for each genotype of the core collection of *M. truncatula* (n=155)

Table S5 Estimation of the heritability of the ecophysiological parameters

Table S6 GWAS output for the ecophysiological parameters

Table S7 List of Gene IDs corresponding to the significant SNPs in the GWAS for each ecophysiological traits and their corresponding annotation, GO id and GO term

Table S8 Richness, alpha- and beta-diversity indices of the rhizosphere bacterial communities associated with the *Medicago truncatula* core collection

Table S9 Filtered and normalized occurrence table including all samples (n=435) used in alpha- and beta-diversity analyses, bacterial composition, and functional prediction

Table S10 Taxonomic affiliation, relative abundance, and properties in the co-occurrence network of the 150 OTU found significantly correlated in the co-occurrence network analysis

Table S11 PERMANOVA results based on Bray-Curtis distances

Table S12 Plant genotype effect on bacterial composition at Phylum and Class levels and on KEGG categories

Table S13 Redundancy analysis on microbial data and ecophysiological for the core collection of

M. truncatula

Table S14 List of the candidate OTU, which are found as major predictor of three plant phenotypic variables and their significantly correlated OTU

Table S15 Potential enzyme classification that are significantly more and less abundant for the OTU positively and negatively linked to the three plant phenotypic variables analyzed in random forest in comparison to the rest of the bacterial OTU and their associated KEGG categories and pathways

Table S16 Estimation of the heritability for the 900 most abundant OTUs

Table S17 Global GWAS output for the abundance of the bacterial OTUs (OTUs predicting plant ecophysiological traits, OTUs in the cooccurrence network and the top 157 most abundant OTUs) and for the two first principal component axes of the ordination plot representing the bacterial community (using the top 10% more abundant OTU))

Table S18 List of Gene IDs corresponding to the significant SNPs in the GWAS for each analysis of VIP OTU group predicting ecophysiological traits and their corresponding annotation, GO id and GO term

Table S19 List of Gene IDs corresponding to the significant SNPs in the GWAS for each OTU group (cooccurrence network and top 157 most abundant ones), their corresponding annotation, GO id and GO term, and topGO output

Notes S1 Zip file containing data sets and R scripts used to produce the analyses and figures

Methods S1 Detailed materials and Methods

Plant material and culture conditions

Seeds of the 192 accessions used in this study were obtained from the *Medicago truncatula* biological resource center GAMéT (UMR Agap, Montpellier, France). Because of either a lack of SNP information, low germination rates, or early flowering time for some accessions, 155 accessions were characterized and used in the GWAS study (Table **S1**). *Medicago truncatula* seeds were scarified and surface sterilized (Mougel *et al.*, 2006), vernalized at 4°C during 48 h and germinated on 0.7% (w/v) water agar plates at 25°C in the dark. A single germinated seed of each *Medicago truncatula* genotype was sown in a pot containing 1 kg of a silty-clayey soil (Mas d'Imbert, France) which hosts many symbiotic microbes (Offre *et al.*, 2007). The physicochemical characteristics of this soil were determined as: 11% sand, 51% silt, 38% clay, pH= 8, 14.5 g.kg^{-1} of organic carbon, 1 g.kg^{-1} of N, 0.018 g.kg^{-1} of NO_3, 0.002 g.kg^{-1} of NH_4 and 1 g.kg^{-1} of P_2O_5. Twelve biological replicates for each accession were grown in a randomized design (Table **S2**). The plants were cultivated up to the end of their vegetative period (see Plant growth section) at a density of 45 plants.m^{-2} in a greenhouse, under a photoperiod of 14 hours and a temperature of 25/19°C (day/night). Supplementary artificial light was supplied with sodium lamps (MACS 400 W; Mazda, Dijon, France) to complement photosynthetically active radiation. To maximize interactions between *Medicago truncatula* and some microbial populations of its microbiota, including but not exclusively its nitrogen fixing bacteria and arbuscular mycorrhiza fungi symbionts, that could improve plant nutrition and growth, plants were only watered with osmotic water (pH=7). An automatic watering scheme considering the weight of a control pot was used to maintain soil moisture at 45 % of its maximum soil water-holding capacity around-the-clock, and a manual control watering was also performed every week.

Plant phenotyping

Plant growth, N content and nodulation score

At three sampling dates during the vegetative developmental stage (488, 734 and 848 degree-days after sowing, or respectively 28, 42 and 49 days after sowing), three plants per accession were harvested. The level of nodulation was evaluated using a visual scale (Moreau *et al.*, 2008).

Shoot and root biomasses were determined after drying at 80°C during 48 h. Then, total nitrogen (N) content was measured using a CHN (Carbon Hydrogen Nitrogen) analyzer (Carlo Erba, Val de Reuil, France). At the third harvest data point (848 degree-days after sowing) the same plants were used for both plant phenotypic and rhizosphere bacterial composition analyses. Furthermore, a different plant batch of three plants per accession was used to estimate leaf area of *Medicago truncatula* every week from 19 to 54 days after sowing. To do so, a non-invasive monitoring setup was used as previously described (Moreau *et al.*, 2009). The pictures were analyzed using a software for image analysis to estimate the projected leaf area (Visilog 5.4; Noesis, Les Ulis, France). Projected leaf area was determined by comparing pixel value for each plant to pixel value of a square standard with a known area which was placed close to the shoot part for every picture. Nodulation was visually assessed, using a qualitative scale (Moreau *et al.*, 2008). Finally, dry biomasses, N content and leaf area were used to calculate plant ecophysiological parameters.

Plant C and N nutritional strategies using an ecophysiological framework

The different plant accessions could employ contrasted carbon (C) and nitrogen (N) nutritional strategies to reach similar/different growth. An ecophysiological framework (Moreau *et al.*, 2012) was used to study these different plant nutritional strategies. This framework links four integrative variables (leaf area, total dry weight, root dry weight and total amount of N in plant) with four intermediate parameters: the radiation use efficiency (RUE) for biomass production, the root to total biomass ratio (RTR), the plant-specific N uptake (SNU) and the conversion factor of N to leaf area (NLA) (Moreau *et al.*, 2012).

Nitrogen nutrition index

A nitrogen nutrition index (NNI) was calculated to estimate the plant N nutrition level. The N nutrition level was considered as optimal when the NNI was close to 1 and a N-deficiency or a N-excess nutrition level was revealed by respectively a NNI lower or higher than 1. The NNI represents the ratio between shoot N concentration and the ‘‘critical shoot N concentration’’ ($\%N_{CS}$) which was defined by the critical dilution curve, as follows: $\%N_{CS} = 8.1 \times SDW^{-0.1}$, where SDW was shoot dry weight (g) (Gastal & Lemaire, 2002; Moreau *et al.*, 2008).

Bacterial diversity, composition, functional prediction, and potential interactions

Isolation, extraction, and purification of bacterial DNA from rhizosphere soil

DNA was extracted from rhizosphere soil as previously described by Mougel and collaborators (Mougel *et al.*, 2006). The rhizosphere soil was obtained after manually separating the root system with adhering soil from the container and by washing the root system under agitation (vortex at 30 hertz for 1 minute) in 50 ml of sterile 0.025 M K_2SO_4 solution. The root system was discarded (for biomass, N content and microbial analyses) and the soil was collected after centrifugation (Zancarini *et al.*,2012). Three replicates per accession were used. Each sample was weighed, frozen in liquid nitrogen and conserved at -80°C.The DNA extraction procedure was based on chemical and mechanical extraction, and DNA preparations were quantified as previously described by Mougel and collaborators (Mougel *et al.*, 2006).

Polymerase Chain Reaction (PCR) and sequencing

The variable region V4 of the 16S rRNA gene was amplified using the F479 and R888 primers (Terrat *et al.*, 2015) and sequenced using Illumina MiSeq sequencing technology by GenoScreen (Lille, France, https://www.genoscreen.fr/fr/).

Bioinformatics sequence analysis

Bioinformatic analyses were done using the GnS-PIPE, now renamed BIOCOM-PIPE (Terrat *et al.*, 2012; Djemiel *et al.*, 2020). Chosen parameters for each step can be found in Methods **S1** Table **1** and the details of all steps have been already described previously (Djemiel *et al.*, 2020). After preprocessing, alignment and clustering of reads at 95% of similarity, a filtering step was then carried out to perform a check on all single-singletons (reads detected only once and not clustered) to eliminate PCR chimeras and large sequencing errors produced by the PCR step, based on the quality of their taxonomic assignments. The number of high-quality reads for each sample was normalized (10,000 high-quality reads for each sample) by random selection to allow efficient comparison of the data sets and avoid biased community comparisons. Then, as the analysis of bacterial community richness relies on the construction of similarity clusters (called OTU), we chose here to use OTU to examine the distribution of 16S rRNA gene sequences in our datasets. However, there is no single best definition of 'species', '*genus*' when this approach is

used, because of controversy about thresholds of similarity allowing clear differentiation of taxonomic units (Schloss & Westcott, 2011). Moreover, a study regarding the diversity of bacterial genomes demonstrated that when the standard threshold of 97% is used, some species can fall to different OTU due to intragenomic or intraspecific differences (Větrovský & Baldrian, 2013). So, we decided to apply the 95% threshold of sequence similarity, usually considered as the '*genus*' level. This clustering was realized with BIOCOM-PIPE. Finally, a global contingency table of OTUs was obtained with the samples in lines and OTUs in columns, indicating the number of reads in each OTU for all samples. The taxonomy of each OTU was determined based on the taxonomy of all reads encompassed in the OTU. More precisely, a taxonomy is given for an OTU if it is composed of more than 90% of reads with the same taxonomy. All raw data sets are publicly available in the European Nucleotide Archive (ENA) of European Molecular Biology Laboratory's European Bioinformatics Institute (EMBL-EBI) database system under project accession no PRJEB25849.

Between-samples normalization

The OTUs with counts lower than 41 over all the samples were filtered out. The threshold of 41 was determined thanks to the following procedure: we calculated for an increasing threshold (from 1 to 150) the number of OTUs with total counts over all samples inferior to this threshold. We selected the threshold for which the number of OTUs does not increase if we augment this threshold of 1. One sample was removed because of its too low-depth (Weiss *et al.*, 2017) and five outsiders were also removed.

For the analysis of the alpha- and beta-diversity, the bacterial composition and functional prediction, we first performed a total count between-sample normalization to correct for the different sequencing depth. Each sample was scaled by a size factor calculated as the ratio between the total number of counts in this sample and the mean of total counts across all samples (Dillies *et al.*, 2013). A total of 435 samples and 15,617 OTUs were finally kept for subsequent analyses (Fig. **S1**, Table **S9**).

For the GWAS and the random forests, the number of reads in each OTU was first summed for the three replicates of each plant genotype. Then, to correct for the different sequencing depth a between-sample normalization was performed as it has been described above for all the

samples and replicates. A total of 155 samples (*i.e.* 155 plant genotypes) were finally kept for subsequent analyses (Fig. **S1**). For the GWAS, the different OTUs assessed were submitted to a square root transformation for a better fit to a gaussian distribution and analyzed. For the random forests, the rare OTUs were discarded (<0.01%) to drastically decrease the number of variables (from 15,617 to 913 OTUs that represent only 6% of the OTU number but 83% of the reads) removing the less informative variables (Fig. **S1**).

Alpha- and beta-diversity

Alpha diversity indices were calculated using the package vegan (version 2.5-7) (Oksanen *et al.*, 2020). While we used the non-filtered and non-normalized OTU occurrence data set for the richness, the Chao1 and the rarefaction curves (Methods **S1** Fig. **1**), we used the filtered and normalized OTU occurrence data set for the Shannon, Pielou's evenness and inverse Simpson (Fig. **S1**). Indeed, as Chao1 is a species richness prediction using the number of singletons and doubletons, Chao1 should not be calculated on filtered or normalized data (Deng *et al.*, 2024). Richness represents the OTU number (at 95% similarity thresholds), Chao1 estimates the total richness, and Pielou's evenness provides information about the equity in OTU abundance.

The beta-diversity was estimated using both Bray-Curtis and Sørensen distances at the OTU level to measure the variation among samples and plant genotypes for the bacterial communities. Bray-Curtis dissimilarities were calculated on the filtered and normalized OTU occurrence data set using the package vegan. Then, a PERmutational Multivariate Analysis Of VAriance (PERMANOVA) test was run using the adonis function, included in the vegan package, to test the effect of the plant genotype on the beta-diversity of the rhizosphere bacterial communities of the core collection of *M. truncatula*. Following the procedure described by Baselga (Baselga, 2010), beta-diversity was also estimated based on presence-absence data using pairwise Sørensen dissimilarities in order to obtain the total beta-diversity and its partitioning in terms of turnover (OTU replacement) and nestedness (OTU loss or gain) using the betapart package (Baselga & Orme, 2012).

Functional prediction

Functional prediction of the bacterial communities associated to the core collection of *M. truncatula* was assessed using PICRUSt2 (Phylogenetic Investigation of Communities by Reconstruction of Unobserved States) version 2.3.0-b (Langille *et al.*, 2013; Douglas *et al.*, 2020). To do so, the normalized occurrence table has been used into the picrust2_pipeline.py script to generate predicted microbial enzyme classification (EC) and pathway abundance. MetaCyc pathway identifiers were then mapped to their respective pathway names using the picrust2 add_descriptions.py script. Correspondence between EC and Kyoto Encyclopedia of Genes and Genomes (KEGG) pathway and categories were also downloaded from https://www.genome.jp/kegg-bin/get_htext#B3 (version of the October 14, 2020) in order to be able to group EC per KEGG categories. Finally, the predicted microbial enzyme classification (EC) data set was rarefied.

Co-occurrences network analysis

To identify potential associations among the core rhizosphere bacterial OTUs, we calculated Spearman correlations using the SparCC method (Sparse Correlations for Compositional data (Friedman & Alm, 2012), available at https://bitbucket.org/yonatanf/sparcc, yonatanf-sparcc-3aff6141c3f1, using 500 iterations and 500 bootstraps) among the normalized relative abundance levels of rhizosphere bacterial OTUs which are present in all the samples (n = 435). Within the 15,617 OTUs, 482 OTUs were shared among all the samples. The resulting matrix of correlation coefficients was parsed in R software using the function "exportNetworkToCytoscape" in the WGCNA package (Langfelder & Horvath, 2008). Only significant correlations (pseudo P-values ≤ 0.05 based on 500 bootstraps) with an absolute correlation magnitude ≥ 0.5 were considered for the network display using Cytoscape software version 3.9.1 (Shannon *et al.*, 2003). The network was visualized using the edge-weighted spring embedded layout algorithm in Cytoscape without forcing by correlation weight values. The nodes represented rhizosphere bacterial OTUs and were connected by edges (positive or negative correlations). The resulting network had 394 edges, with 150 distinct bacterial OTUs. Network properties (e.g. density, diameter, mean distance, transitivity, assortativity, betweenness, eigen-centrality and cliques) and the 1000 random networks were calculated using igraph package (Csardi & Nepusz, 2006). Groups of OTUs were

defined using the betweenness edge clustering method and hubs were pinpointed based on both their degree and betweenness centrality.

Random Forests

We conducted three regression random forests (RF) (Breiman, 2001) analyses to identify the major OTU predictors for three plant phenotypic variables (total dry biomass at 848 degree-days after sowing (TDW VS3), RTR and SNU). To decrease the number of OTU used in the RF analyses, we removed rare OTUs (<0.01%) as described previously in the Between-samples normalization section. These analyses were conducted using the MUVR package of the R software version 0.0.973 (Shi *et al.*, 2019). The MUVR algorithm achieves a minimal feature selection by performing recursive variable elimination in a repeated double cross-validation procedure and improves then predictive performance minimizing over-fitting and false positives. We set the parameters as following: number of repetitions (nRep) = 40, number of outer cross-validation segment (nOuter) = 8, variable ratio parameter that governs the proportion of variables kept for iteration of the recursive variable elimination in the inner loop (varRatio) = 0.8 and we looked at the all-relevant model (max) that included predictors with redundant but not erroneous information. The statistical significance of the models was assessed with 100 permutations of the plant phenotypic variable.

Estimates of genetic variance and heritability

Heritability (h^2) estimates the proportion of phenotypic variance that is due to genetic variance. Genetic variance was estimated fitting the linear mixed model $Yij = Gi + eij$, where Yij is the vector of normalized count for each OTU for each genotype i and random repeat k using the R package lme4 version 1.1-31 (Bates *et al.*, 2015)). Then, h^2 was calculated as genetic variance (var_G) divided by the sum of genetic variance and the error variance ($var_G + var_{error}/n$), where n=3 is the total number of replicates in our random design. We considered as heritable a variable with significant non-zero genetic variance (Pvalue of the likelihood ratio test for the genetic effect lower than 0.05) and h^2 higher than 0.25. Furthermore, as we used the average of the three replicates for each heritable trait (i.e. ecophysiological variables or OTUs) in the following GWAS analyzes, we

checked the variation within the replicates by calculating their coefficients of variation (Fig. **S2**; Fig. **S7**), and removed traits for which the median of their coefficients of variation from the different plant genotypes was greater than 0.6 (i.e. OTU15, OTU101 and OTU189).

Procedure for GWAS

SNP (Single Nucleotide Polymorphism) data were obtained by Illumina sequencing from the *Medicago truncatula* HapMap project (Stanton-Geddes *et al.*, 2013) and were filtered and imputed as described by Le Signor and collaborators (Le Signor *et al.*, 2017). The GEMMA (Genome-wide Efficient Mixed Model Association algorithm) software v0.94 (Zhou & Stephens, 2012) was used to test through a standard linear mixed model for marker association with a single phenotype accounting for population stratification (Q) and sample structure (K). Kinship matrix (K) was calculated with all intergenic markers following the GEMMA procedure (centered relatedness matrix was computed). The structure (Q matrix) used was the same as that described by Bonhomme and collaborators (Bonhomme *et al.*, 2014) (two genetic groups). We specify a missing data threshold of 5%. P-values for each SNP were calculated from a likelihood ratio test. The -log10 P-values were exported to generate Manhattan plots (Methods **S1** Fig. **2**) using SNP with *P*-values less than 0.001 and Q-Q plots using a randomly selected set of 10% of all SNPs from the eight chromosomes (R library qqman; R Core Team, 2014 (R Development Core Team, 2014)). Q-Q plots display the calculated SNP *P*-values (*y*-axis) versus the expected uniform distribution of *P*-values, assuming no association between the trait and the SNPs (*x*-axis). SNPs strongly associated with the trait of interest, with low *P*-values, will locate to the upper right end of a Q-Q plot, above the middle line (Benjamini & Hochberg, 1995). The positions of SNPs inside or in the vicinity of genes were found in context files downloaded from https://medicago.legumeinfo.org/. The annotations of listed genes were confirmed with the Mt4.0v1 genome version (http://www.jcvi.org/medicago/). To refine candidate gene selection, a GEMMA P-value cut-off at 10^{-6} was applied to reduce the false-positive rate while retaining minor effects SNPs. The 10^{-6} cut-off was calculated by Bonhomme and collaborators (Bonhomme *et al.*, 2014) based on the estimated number of Linkage Disequilibrium (LD)-blocks in the genome from the same population and genetic data. They found that the number of LD blocks ranged from 29,041 to 55,463,

depending on the block calculation window size (5, 10, and 50 kb). This resulted in significance thresholds between 9.02 10^{-7} and 1.72 10^{-6} for the Bonferroni correction, at a nominal significance level of the α test of 5%. Following these authors, we therefore decided to use a consensus GWAS threshold of $P < 10^{-6}$. Singular Enrichment Analyses (SEA) using an exact Fisher test were done with topGO R package (Alexa & Rahnenfuhrer, Jorg, 2016) using GO term annotations from *Medicago truncatula* v4.0v1(https://jgi.doe.gov, (Tang *et al.*, 2014)).

Multivariate and statistical analyses

All statistical analyses were performed and figures were plotted using the statistical software R 4.1.1 (R Development Core Team, 2014) within the RStudio 1.4.1717 Integrated Development Environment (RStudio Team, 2020). Only significant differences at P-values < 0.05 were considered. R scripts are available in Notes **S1** together with their required data sets inputs. A list of the acronyms used is available in Table **S3**.

Five groups of plant genotypes were defined using a clustering method for 146 genotypes (due to missing values). To do so, only 13 plant phenotypic variables, which were not highly correlated (|Spearman correlation| < 0.6, Methods **S1** Table **2**), were selected for this analysis (i.e. leaf area at 488 degree-days after sowing (LA VS1), total dry biomass at 488 degree-days after sowing (TDW VS1), root to total dry biomass at 488 degree-days after sowing (RTR VS1), nitrogen ratio at 488 degree-days after sowing (NR VS1), total dry biomass at 734 degree-days after sowing (TDW VS2), root to total dry biomass at 734 degree-days after sowing (RTR VS2), nitrogen ratio at 734 degree-days after sowing (NR VS2), leaf area at 848 degree-days after sowing (LA VS3), total biomass at 848 degree-days after sowing (TDW VS3), nitrogen nutritional index at 848 degree-days after sowing (NNI VS3), RUE, RTR and SNU). We did not keep all the 30 ecophysiological variables measured or calculated, and selected only these 13 variables because some of them were partially redundant. As an example, the leaf biomass, the root biomass and the carbon content (=%carbon*total biomass) were redundant and highly corelated with each other, and with the total biomass (=leaf+root biomasses). So, we chose to keep only the total biomass and root to total biomass ratio (RTR) to not artificially increase the contribution of the biomass variables in discriminating ecophysiological groups of plant. Therefore, only non-redundant phenotypic

information was retained for biological interpretation. Then, euclidean distances were calculated on the centered and scaled plant phenotypic data set. Finally, five groups of plant genotypes were defined using the hclust “ward.D2” linkage method and the cutree function in R software.

Effects of plant genotype and ecophysiological groups on leaf area, plant dry biomasses, plant nitrogen ratio, NNI, RUE, RTR, SNU, NLA and bacterial diversity indices were analyzed using Kruskal-Wallis tests followed by Dunn tests.

Then, using the same approach as Horton and collaborators (Horton *et al.*, 2014), we tested the hypothesis that plant genotype shaped the rhizosphere bacterial community of *Medicago truncatula* using the function envfit of the R package vegan. To do so, after Hellinger transformation, Principal Component Analyses (PCA) were performed on the filtered and normalized OTU dataset (n=435, 155 plant genotypes and their replicates) using the function rda in the package vegan (Oksanen *et al.*, 2020) for all the OTUs, the 50%, 20%, 15%, 10%, 5%, 4%, 3%, 2%, and 1% most heavily sequenced OTUs.

Moreover, we tested if we could explain the variation in the bacterial community composition at the last harvest (848 degree-days after sowing) by the variation in the plant ecophysiological phenotype using a redundancy analysis (RDA). To do so, we selected only the 13 plant phenotypic variables, which were not highly correlated (|Spearman correlation| < 0.6, Methods **S1** Table **2**): LA VS1, TDW VS1, RTR VS1, NR VS1, TDW VS2, RTR VS2, NR VS2, LA VS3, TDW VS3, NNI VS3, RUE, RTR and SNU and used rda and anova.cca functions in the package vegan (Oksanen *et al.*, 2020). We also assessed the marginal effects of the terms in the model using by=”margin”.

Finally, in order to test if some functions were potentially more and less abundant for the candidate OTUs identified by the RF analysis versus the rest of the bacterial communities, we first defined the list of OTUs that were significantly correlated to the candidate OTUs. Indeed, RF considers all the variables independently (here OTUs) and selects only the most relevant variables that can predict the response variable (here plant phenotype). It is important for us to select all the relevant candidate OTUs (*i.e.* OTUs identified by the RF and OTU that are significantly correlated to them). To do so, the cor and corr.test functions were used in R software using the Spearman method and Bonferroni correction. Therefore, for each plant phenotypic variable, an extended list of candidate OTUs was defined, from which OTUs with a nearest-sequenced taxon

index (NSTI) value superior to 2 were then removed (249, 156, 233, and 138 OTU for TDW VS3, RDW VS3, RTR and SNU, respectively). Then, we divided these lists of OTUs in two: OTU positively and negatively correlated to each of the plant phenotypic variables. Next, a two-sided t-test was applied using function getPvalues of topGO R package version 2.44.0 (Alexa & Rahnenfuhrer, Jorg, 2016) on the EC_predicted.tsv output file from PICRUSt2, for which both rare and non-well characterized OTUs (NSTI>2) were previously removed, in order to identify EC that were potentially differentially abundant between the extended candidate OTUs list and the rest of the non-rare OTUs. Finally, KEGG categories and pathways associated to the significantly differentially abundant EC were defined (using EC KEGG correspondence table downloaded from https://www.genome.jp/kegg-bin/get_htext#B3, version of the October 14, 2020).

Methods S1 Fig.1 Rarecurves

Rarefaction curves were calculated using the vegan package. Each line represents the rarecurve for each sample (n=435).

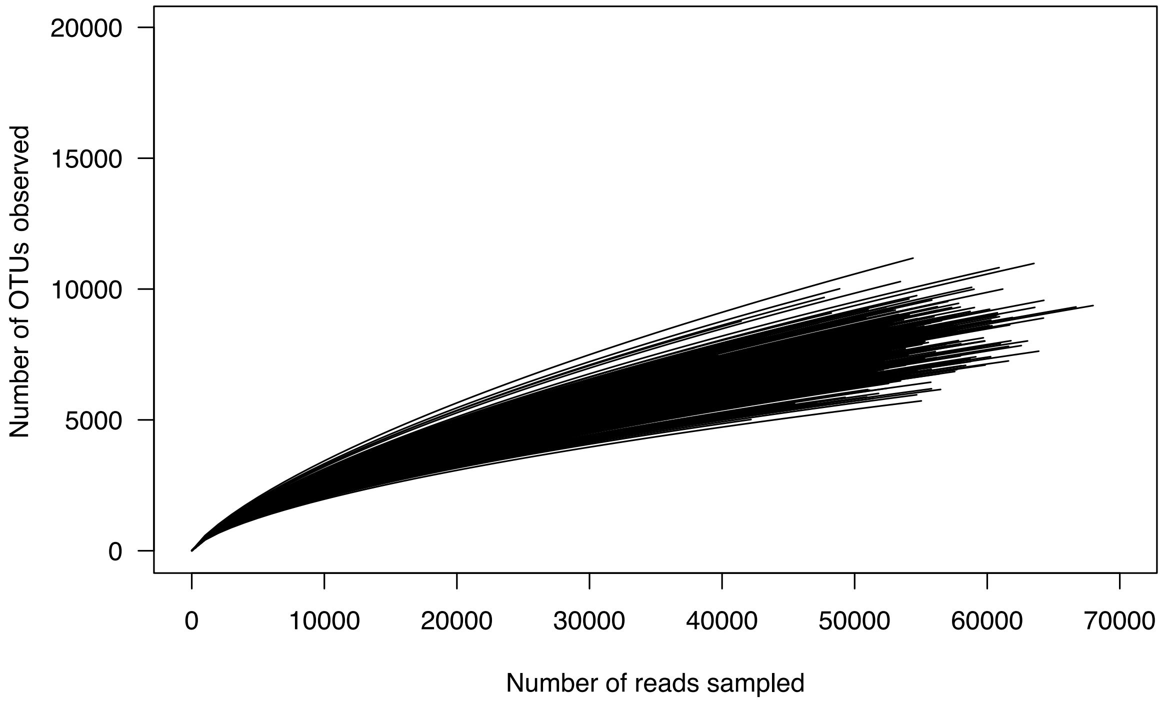

Methods S1 Fig. 2 QQplots for all the heritable OTU analyzed in GWAS

Q-Q plots display the calculated SNP P-values (y-axis) versus the expected uniform distribution of P-values, assuming no association between the trait and the Single Nucleotide Polymorphisms (SNPs) (x-axis). SNPs strongly associated with the trait of interest, with low P-values, will locate to the upper right end of a Q-Q plot, above the middle line. Only a randomly selected set of 10% of all SNPs from the eight chromosomes were represented on these Q-Q plots.

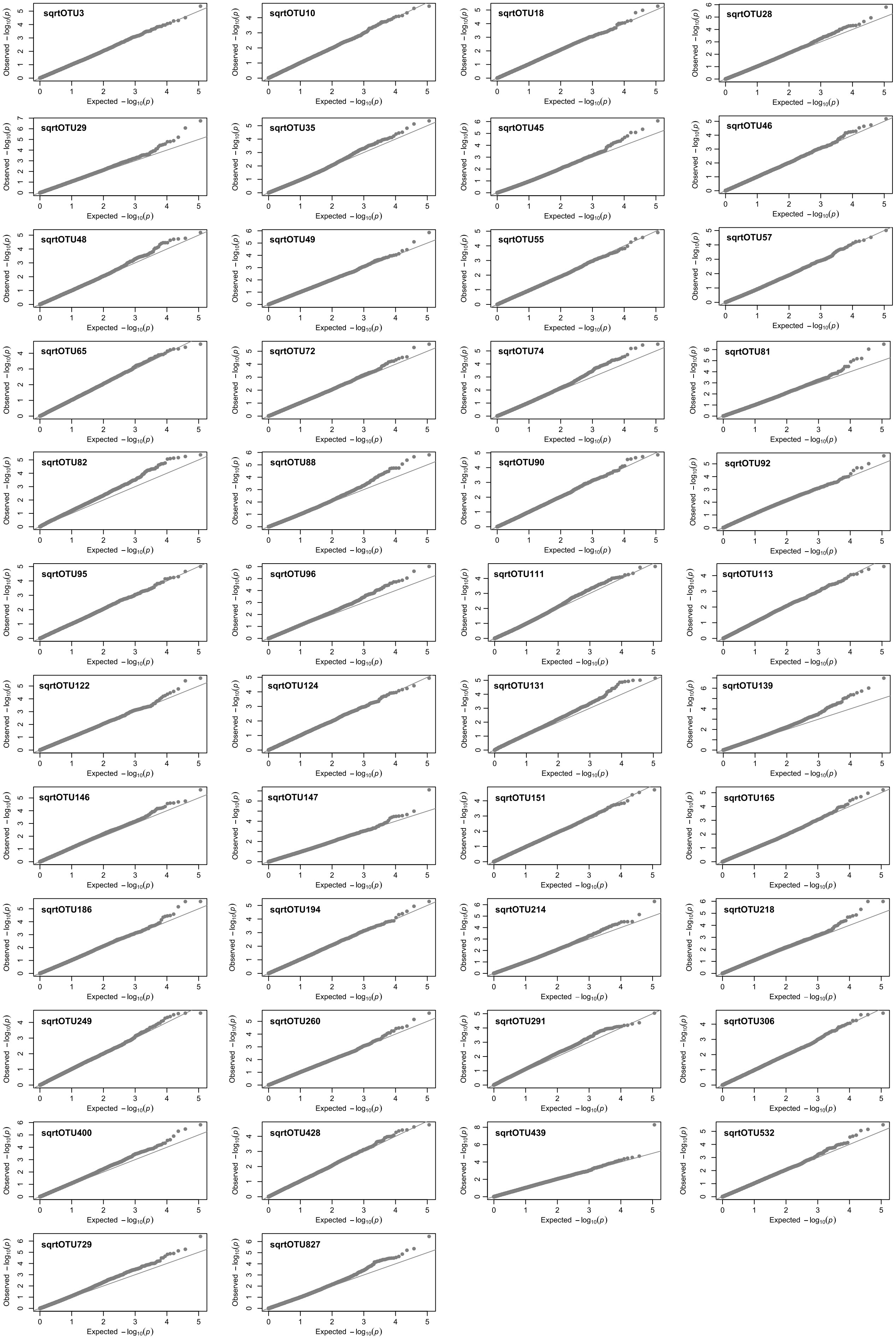
sqrtOTU3
sqrtOTU10
sqrtOTU18
sqrtOTU28
sqrtOTU29
sqrtOTU35
sqrtOTU45
sqrtOTU46
sqrtOTU48
sqrtOTU49
sqrtOTU55
sqrtOTU57
sqrtOTU65
sqrtOTU72
sqrtOTU74
sqrtOTU81
sqrtOTU82
sqrtOTU88
sqrtOTU90
sqrtOTU92
sqrtOTU95
sqrtOTU96
sqrtOTU111
sqrtOTU113
sqrtOTU122
sqrtOTU124
sqrtOTU131
sqrtOTU139
sqrtOTU146
sqrtOTU147
sqrtOTU151
sqrtOTU165
sqrtOTU186
sqrtOTU194
sqrtOTU214
sqrtOTU218
sqrtOTU249
sqrtOTU260
sqrtOTU291
sqrtOTU306
sqrtOTU400
sqrtOTU428
sqrtOTU439
sqrtOTU532
sqrtOTU729
sqrtOTU827
Observed −log10(p)
Expected −log10(p)

Methods S1 Table 1 Bioinformatic parameters and databases used in the metabarcoding analysis of 16S rRNA gene sequences

STEP	PARAMETER DESCRIPTION	PARAMETER(S)
**PREPROCESSING**	Minimum length threshold	350
	Number of ambiguities tolerated	0
	Detection of proximal primer sequence	Complete and perfect
	Detection of distal primer sequence	Complete and perfect
**CLUSTERING**	Chosen level of similarity (%)	95
	Ignoring differences in homopolymer lengths	Yes
**FILTERING**	Chosen clustering similarity threshold	95
	Used taxonomic database	SILVA (r114)
	Chosen taxonomic level	Phylum
	Similarity or confidence threshold (%)	90
**HOMOGENIZATION**	High-quality reads kept for each sample	10,000
**GLOBAL ANALYSIS**	Chosen level of similarity (%)	95
	Ignoring differences in homopolymer lengths	Yes

New Phytologist

Methods S1 Table 2 Spearman correlations among the plant phenotypic variables measured and calculated for the core collection of *M. truncatula*

Spearman correlations were calculated between variables using the cor function in R. The p-values were estimated using the corr.test in the R package psych (Best & Roberts, 1975; 'Wiley Series in Probability and Statistics', 2015).

Abbreviations: LA: Leaf Area (cm^2) ; SDW: Shoot Dry Weight (g); RDW: Root Dry Weight (g); TDW: Total Dry Weight (g); NC: Nitrogen content (g); NR: Nitrogen Ratio (%); NNI: Nitrogen Nutrition Index; NLA: conversion factor of Nitrogen to Leaf Area (cm^2 of leaf g-1 of N); RUE: Radiation Use Efficiency (g of total dry biomass MJ-1 of intercepted PAR); RTR: Root to Total biomass Ratio, SNU: plant-Specific Nitrogen Uptake (g N g-1 of belowground dry biomass day-1); VS1: Vegetative developmental Stage 1 (488 degree-days or 28 days after sowing); VS2: Vegetative developmental Stage 2 (734 degree-days or 42 days after sowing); VS3: Vegetative developmental Stage 3 (848 degree-days or 49 days after sowing).

	LA_VS1	SDW_VS1	RDW_VS1	TDW_VS1	RTR_VS1	NR_VS1	NC_VS1	LA_VS2	SDW_VS2	RDW_VS2	TDW_VS2	RTR_VS2	NR_VS2	NC_VS2	LA_VS3	SDW_VS3	RDW_VS3	TDW_VS3	RTR_VS3	NR_VS3	NC_VS3	NNI_VS3	RUE	RTR	SNU	NLA
LA_VS1	1	0.52	0.51	0.53	0.10	0.05	0.53	0.44	0.39	0.40	0.41	0.06	-0.04	0.41	0.35	0.20	0.30	0.24	0.14	-0.05	0.25	0.10	-0.29	0.12	-0.32	0.08
SDW_VS1	0.52	1	0.86	0.98	0.00	0.14	0.98	0.22	0.52	0.55	0.54	0.06	-0.11	0.52	0.15	0.25	0.35	0.30	0.15	-0.12	0.28	-0.02	-0.13	0.11	-0.58	0.02
RDW_VS1	0.51	0.86	1	0.95	0.44	0.06	0.93	0.10	0.41	0.45	0.44	0.06	-0.09	0.42	0.07	0.19	0.29	0.25	0.17	-0.16	0.22	-0.02	-0.05	0.05	-0.56	-0.02
TDW_VS1	0.53	0.98	0.95	1	0.17	0.11	0.99	0.18	0.49	0.53	0.51	0.07	-0.10	0.49	0.12	0.23	0.34	0.29	0.17	-0.14	0.26	-0.02	-0.10	0.11	-0.59	0.01
RTR_VS1	0.10	0.00	0.44	0.17	1	-0.17	0.14	-0.18	-0.10	-0.05	-0.09	0.03	0.00	-0.09	-0.15	-0.09	-0.07	-0.08	0.06	-0.10	-0.11	-0.06	0.08	-0.10	-0.13	-0.05
NR_VS1	0.05	0.14	0.06	0.11	-0.17	1	0.24	0.07	0.11	0.13	0.12	0.03	0.22	0.17	0.05	0.08	0.03	0.06	-0.03	0.07	0.10	0.13	-0.05	-0.01	-0.07	0.06
NC_VS1	0.53	0.98	0.93	0.99	0.14	0.24	1	0.18	0.49	0.53	0.52	0.08	-0.07	0.50	0.12	0.23	0.33	0.28	0.16	-0.11	0.27	0.01	-0.11	0.10	-0.59	0.01
LA_VS2	0.44	0.22	0.10	0.18	-0.18	0.07	0.18	1	0.39	0.34	0.39	-0.03	0.01	0.40	0.87	0.35	0.30	0.34	-0.05	0.00	0.37	0.09	-0.60	-0.03	-0.05	0.62
SDW_VS2	0.39	0.52	0.41	0.49	-0.10	0.11	0.49	0.39	1	0.84	0.98	-0.22	-0.12	0.96	0.35	0.51	0.46	0.52	-0.08	-0.19	0.49	-0.05	-0.04	-0.09	-0.44	-0.03
RDW_VS2	0.40	0.55	0.45	0.53	-0.05	0.13	0.53	0.34	0.84	1	0.92	0.29	-0.23	0.87	0.28	0.41	0.47	0.45	0.07	-0.15	0.43	-0.01	-0.05	0.05	-0.61	-0.05
TDW_VS2	0.41	0.54	0.44	0.51	-0.09	0.12	0.52	0.39	0.98	0.92	1	-0.06	-0.16	0.97	0.34	0.50	0.48	0.52	-0.03	-0.18	0.49	-0.04	-0.04	-0.05	-0.51	-0.04
RTR_VS2	0.06	0.06	0.06	0.07	0.03	0.03	0.08	-0.03	-0.22	0.29	-0.06	1	-0.22	-0.10	-0.09	-0.09	0.08	-0.04	0.24	0.08	-0.02	0.12	-0.01	0.26	-0.25	-0.06
NR_VS2	-0.04	-0.11	-0.09	-0.10	0.00	0.22	-0.07	0.01	-0.12	-0.23	-0.16	-0.22	1	0.06	0.00	-0.02	-0.10	-0.04	-0.06	0.31	0.05	0.28	0.00	-0.07	0.24	-0.08
NC_VS2	0.41	0.52	0.42	0.49	-0.09	0.17	0.50	0.40	0.96	0.87	0.97	-0.10	0.06	1	0.34	0.50	0.46	0.52	-0.05	-0.10	0.52	0.05	-0.03	-0.06	-0.45	-0.07
LA_VS3	0.35	0.15	0.07	0.12	-0.15	0.05	0.12	0.87	0.35	0.28	0.34	-0.09	0.00	0.34	1	0.46	0.33	0.44	-0.18	-0.06	0.45	0.04	-0.51	-0.16	0.09	0.66
SDW_VS3	0.20	0.25	0.19	0.23	-0.09	0.08	0.23	0.35	0.51	0.41	0.50	-0.09	-0.02	0.50	0.46	1	0.74	0.97	-0.35	-0.29	0.94	-0.10	0.37	-0.29	0.32	-0.23
RDW_VS3	0.30	0.35	0.29	0.34	-0.07	0.03	0.33	0.30	0.46	0.47	0.48	0.08	-0.10	0.46	0.33	0.74	1	0.87	0.31	-0.35	0.82	-0.10	0.37	0.36	0.04	-0.25
TDW_VS3	0.24	0.30	0.25	0.29	-0.08	0.06	0.28	0.34	0.52	0.45	0.52	-0.04	-0.04	0.52	0.44	0.97	0.87	1	-0.15	-0.32	0.96	-0.09	0.40	-0.09	0.24	-0.26
RTR_VS3	0.14	0.15	0.17	0.17	0.06	-0.03	0.16	-0.05	-0.08	0.07	-0.03	0.24	-0.06	-0.05	-0.18	-0.35	0.31	-0.15	1	-0.08	-0.17	0.02	-0.01	0.95	-0.41	-0.02
NR_VS3	-0.05	-0.12	-0.16	-0.14	-0.10	0.07	-0.11	0.00	-0.19	-0.15	-0.18	0.08	0.31	-0.10	-0.06	-0.29	-0.35	-0.32	-0.08	1	-0.08	0.85	-0.17	-0.07	0.16	-0.05
NC_VS3	0.25	0.28	0.22	0.26	-0.11	0.10	0.27	0.37	0.49	0.43	0.49	-0.02	0.05	0.52	0.45	0.94	0.82	0.96	-0.17	-0.08	1	0.12	0.36	-0.11	0.30	-0.28
NNI_VS3	0.10	-0.02	-0.02	-0.02	-0.06	0.13	0.01	0.09	-0.05	-0.01	-0.04	0.12	0.28	0.05	0.04	-0.10	-0.10	-0.09	0.02	0.85	0.12	1	-0.10	0.03	0.13	-0.10
RUE	-0.29	-0.13	-0.05	-0.10	0.08	-0.05	-0.11	-0.60	-0.04	-0.05	-0.04	-0.01	0.00	-0.03	-0.51	0.37	0.37	0.40	-0.01	-0.17	0.36	-0.10	1	0.02	0.34	-0.84
RTR	0.12	0.11	0.05	0.11	-0.10	-0.01	0.10	-0.03	-0.09	0.05	-0.05	0.26	-0.07	-0.06	-0.16	-0.29	0.36	-0.09	0.95	-0.07	-0.11	0.03	0.02	1	-0.32	-0.05
SNU	-0.32	-0.58	-0.56	-0.59	-0.13	-0.07	-0.59	-0.05	-0.44	-0.61	-0.51	-0.25	0.24	-0.45	0.09	0.32	0.04	0.24	-0.41	0.16	0.30	0.13	0.34	-0.32	1	-0.18
NLA	0.08	0.02	-0.02	0.01	-0.05	0.06	0.01	0.62	-0.03	-0.05	-0.04	-0.06	-0.08	-0.07	0.66	-0.23	-0.25	-0.26	-0.02	-0.05	-0.28	-0.10	-0.84	-0.05	-0.18	1

New Phytologist

Fig. S1 Diagram representing the main analytical steps for both the plant ecophysiological and the bacterial communities' data and their genetic analyzes outputs

Abbreviations: Ecophysio: ecophysilogical traits; TDW: Total Dry Weight (g); RTR: Root to Total biomass Ratio, SNU: plant-Specific Nitrogen Uptake (g N g-1 of belowground dry biomass day-1); VS3: Vegetative developmental Stage 3 (848 degree-days or 49 days after sowing).

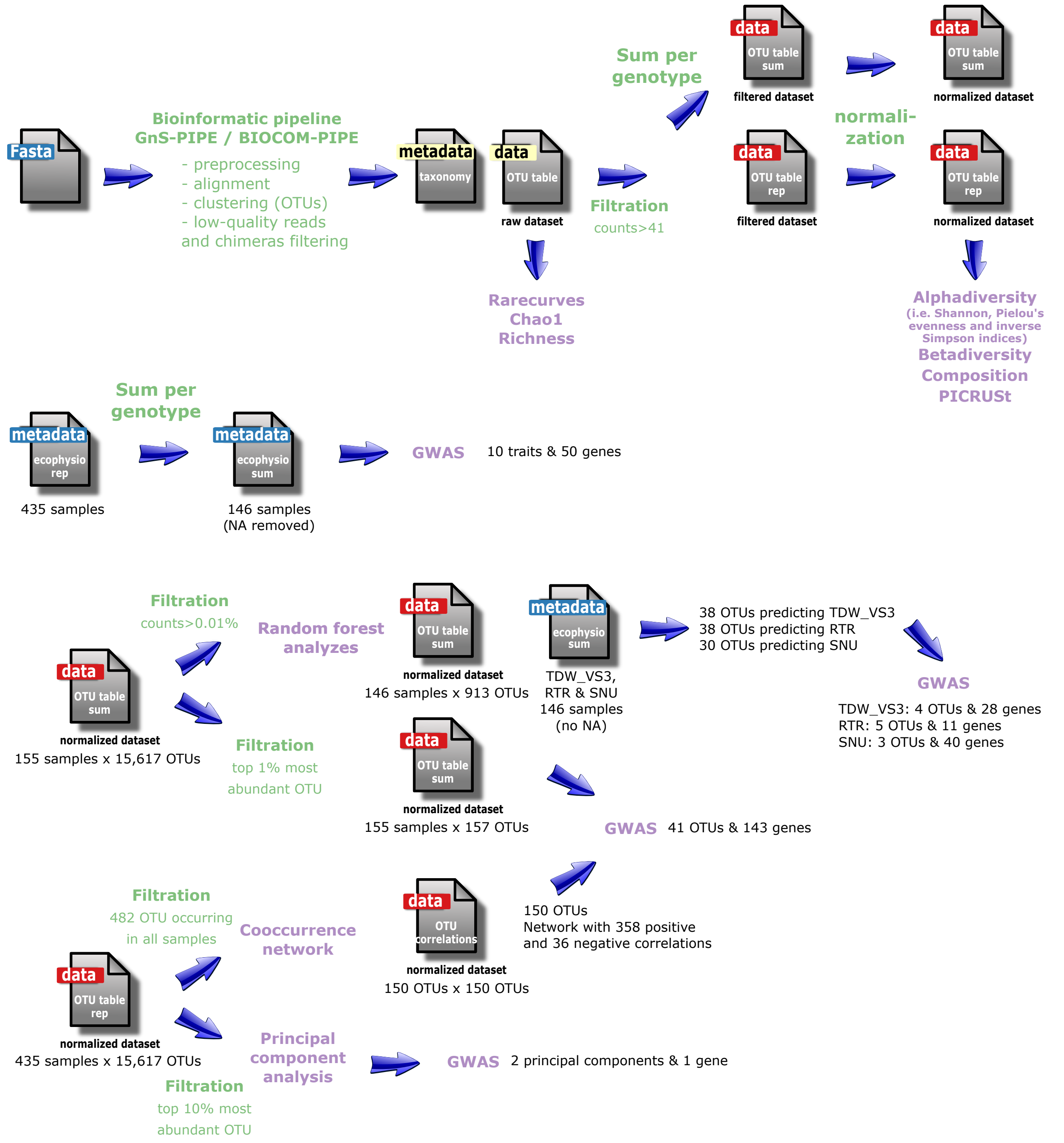

Fig. S2 Coefficients of variation for the ecophysiological traits

Boxplot showing the coefficients of variation for each plant ecophysiological trait. Coefficients of variation were calculated for each ecophysiological trait and each plant genotype as the ratio of the standard deviation to the average for the three replicates (n=146). The black line represents the median value. The lower and upper hinges correspond to the first and third quartiles. The upper and lower whiskers extend from the hinge to the largest and smallest value, respectively (no further than 1.5 time the inter-quartile range). Data beyond the end of the whiskers are plotted individually as black dots.

Abbreviations: LA: Leaf Area (cm²) ; SDW: Shoot Dry Weight (g); RDW: Root Dry Weight (g); RTR: Root to Total biomass Ratio, TDW: Total Dry Weight (g); NC: Nitrogen content (g); NR: Nitrogen Ratio (%); NNI: Nitrogen Nutrition Index; VS1: Vegetative developmental Stage 1 (488 degree-days or 28 days after sowing); VS2: Vegetative developmental Stage 2 (734 degree-days or 42 days after sowing); VS3: Vegetative developmental Stage 3 (848 degree-days or 49 days after sowing).

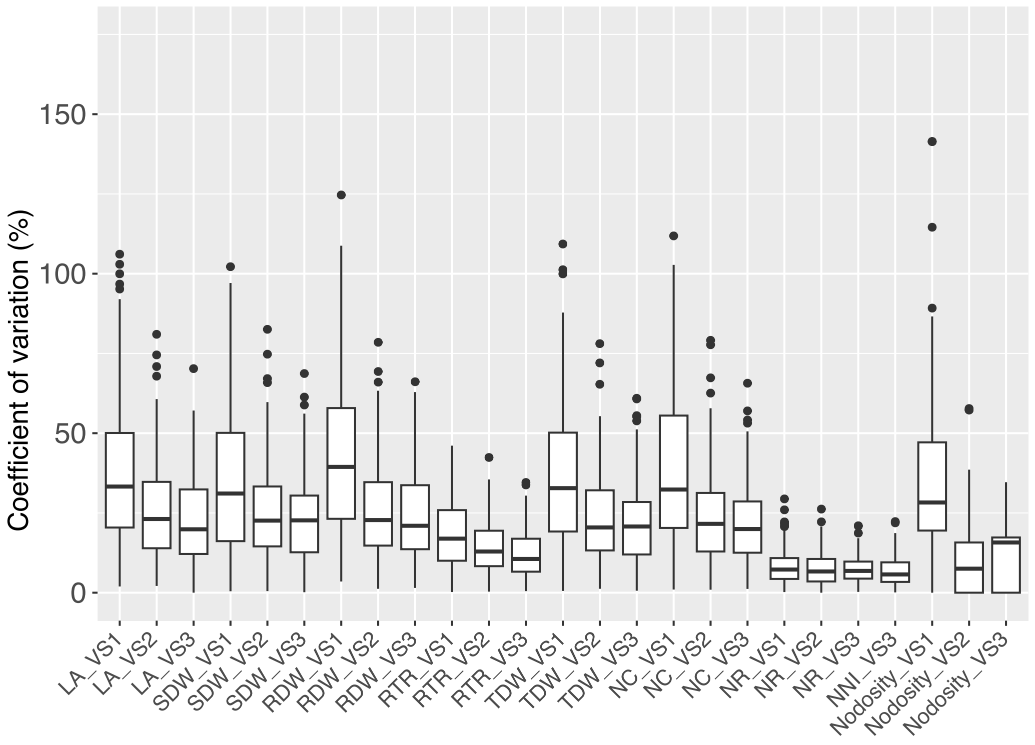

Fig. S3 Potential functions of the rhizosphere bacterial communities associated with the *Medicago truncatula* core collection

Boxplots show occurrences of the potential functions of the rhizosphere bacterial communities associated with the *M. truncatula* core collection (n=435), which were estimated using PICRUSt2 (Langille et al., 2013; Douglas et al., 2020). Microbial enzyme classifications were grouped according to KEGG categories. The black line represents the median value. The lower and upper hinges correspond to the first and third quartiles. The upper and lower whiskers extend from the hinge to the largest and smallest value, respectively (no further than 1.5 time the inter-quartile range). Data beyond the end of the whiskers are plotted individually as black dots.

Abbreviations: Env. Info. Proces.: Environmental_information_processing, KEGG: Kyoto Encyclopedia of Genes and Genomes.

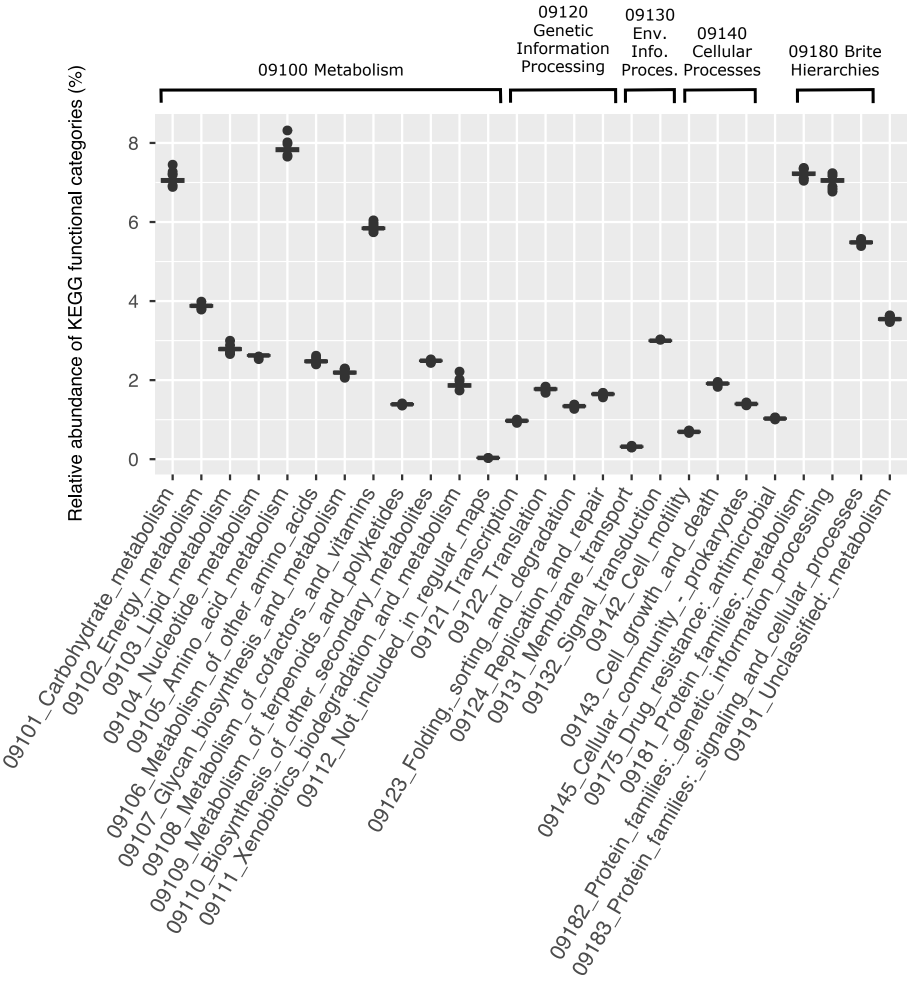

Fig. S4 Co-occurrence network of the *Medicago truncatula* core rhizosphere bacterial community

Spearman correlations were calculated using the SparCC method among the core bacterial OTUs. Only significant correlations (pseudo P-values $\leqslant$ 0.05 based on 500 bootstraps) with an absolute correlation magnitude $\geqslant$ 0.5 were considered for the network display using Cytoscape.

(**a-d**) Co-occurrences network properties compared with 1000 random networks

(**a**) diameter, (**b**) mean distance, (**c**) transitivity and (**d**) assortativity were calculated using igraph package. The values obtained for the co-occurrence network were compared to 1000 random networks with the same number of nodes and an equivalent density of 0.035. Histograms show the distribution of values for the 1000 random networks and the dashed red line represents the value obtained for the co-occurrence network.

(**e**) Hub identification in the network

Hubs were identified considering both degree and betweenness centrality. Filled dots represent the hubs corresponding to the OTUs with degree and betweenness values in the 10% tail for both statistics. Open dots represent the remainder of the core bacterial OTUs.

(**f**) Co-occurrence hubs and OTU identified in the GWAS analyses

The network was visualized using Cytoscape's the edge-weighted spring embedded layout algorithm without forcing by correlation weight values. Nodes represent bacterial OTUs. Brown nodes represent OTU that are hubs in the network (considering only those with degree and betweenness centrality values in the 10% tail of both statistics). Green nodes represent OTU for which plant genetic determinisms were found in the GWAS analyses. Black dashed and solid lines represent negative and positive or correlations, respectively. Line thickness is proportional to the value of the correlations between two nodes; thick and thin lines correspond to high (close to |1|) and low (close to |0.5|) correlations, respectively. The OTU identifier was written on each node. The 14 groups of OTUs were defined using the betweenness edge method.

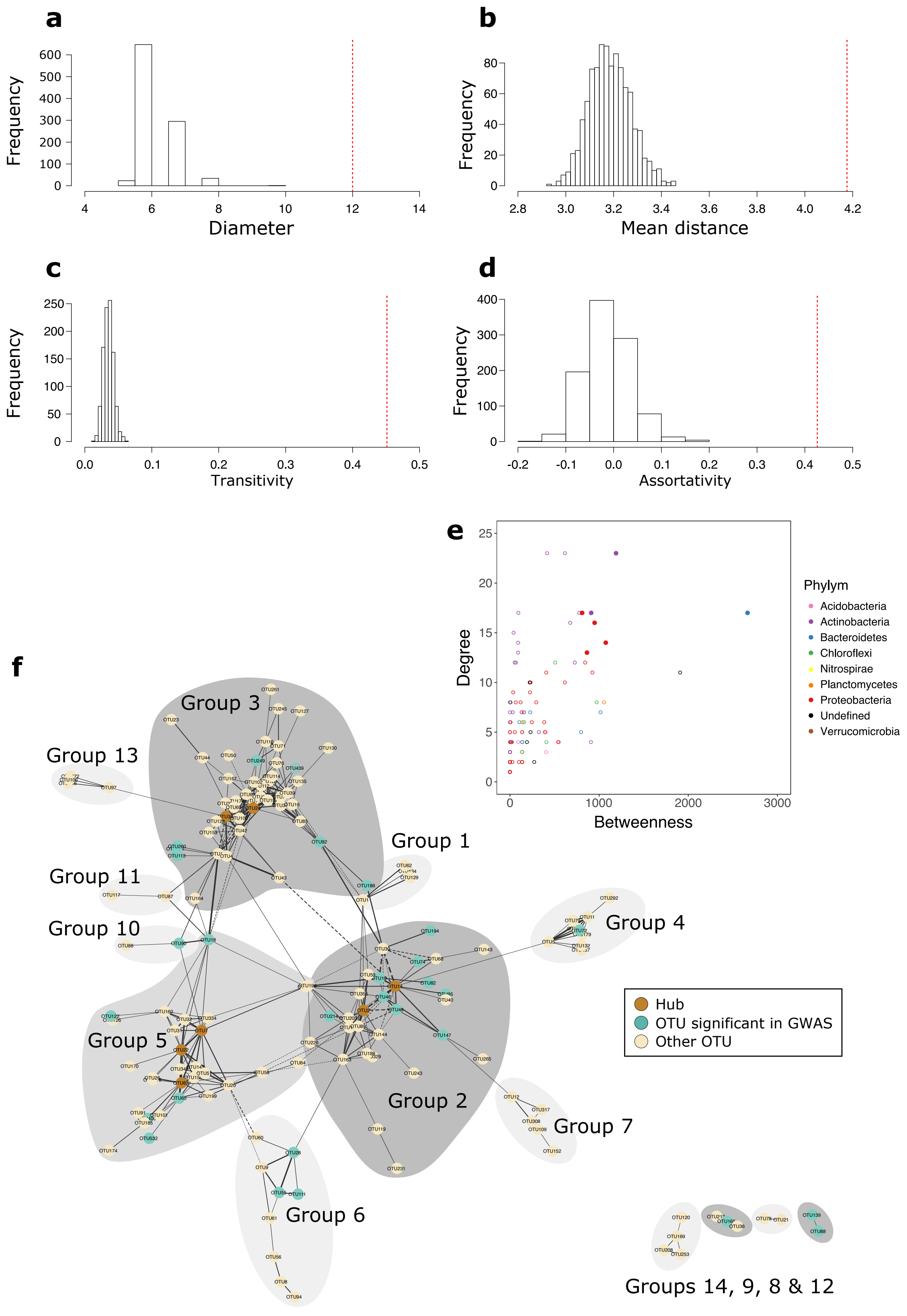
a
Frequency
Diameter
b
Frequency
Mean distance
c
Frequency
Transitivity
d
Frequency
Assortativity
e
Degree
Betweenness
Phylym
Acidobacteria
Actinobacteria
Bacteroidetes
Chloroflexi
Nitrospirae
Planctomycetes
Proteobacteria
Undefined
Verrucomicrobia
f
Group 3
Group 13
Group 1
Group 11
Group 10
Group 4
Group 5
Group 2
Group 7
Group 6
Groups 14, 9, 8 & 12
Hub
OTU significant in GWAS
Other OTU

Fig. S5 Potential KEGG pathways over/under-represented in bacterial OTUs predicting *Medicago truncatula* ecophysiological traits

(**a**), (**c**) and (**e**) represented KEGG pathways over-represented for bacterial OTUs that are major predictors of total biomass at 848 degree-days after sowing (TDW_VS3), root to total ratio (RTR) and specific nitrogen uptake (SNU), respectively; (**b**), (**d**) and (**f**) represented KEGG pathways under-represented for bacterial OTUs that are major predictors of TDW_VS3, RTR and SNU, respectively.

OTUs that were major predictors of the plant ecophysiological traits (TDW VS3, RTR and SNU), were first identified using three regression random forest (RF) analyses. To test whether some functions were potentially more or less abundant for the candidate OTUs compared to the rest of the bacterial communities, we considered the extended list of OTUs that were significantly correlated with the candidate OTUs identified by the random forests. These lists of OTUs were then split in two: OTUs positively and negatively correlated with each of the plant traits. A two-sided t-test using the function getPvalues of topGO R package (Alexa & Rahnenfuhrer, Jorg, 2016) was applied to the EC_predicted.tsv output file from PICRUSt2, for which both rare and non-well characterized OTU (NSTI>2) were previously removed, in order to identify enzyme classifications (EC) that were potentially differentially abundant between the extended candidate OTUs list and the rest of the non-rare OTUs. Finally, KEGG categories and pathways associated with the significantly differentially abundant EC were defined using the EC KEGG correspondence table downloaded from https://www.genome.jp/kegg-bin/get_htext#B3 (version of the October 14, 2020).

Pathways highlighted in dark red represent EC more abundant in the extended candidate OTUs list than in the rest of the OTUs for the OTUs that are positively corelated to the plant ecophysiological traits. Pathways highlighted in light red represent EC less abundant in the extended candidate OTUs list than in the rest of the OTUs for the OTUs that are negatively corelated to the plant phenotypic variable. Pathways highlighted in dark blue represent EC less abundant in the extended candidate OTUs list than in the rest of the OTUs for the OTUs that are positively corelated to the plant phenotypic variable. Pathways highlighted in light blue represent EC more abundant in the extended candidate OTUs list than in the rest of the OTUs for the OTUs that are negatively corelated to the plant phenotypic variable.

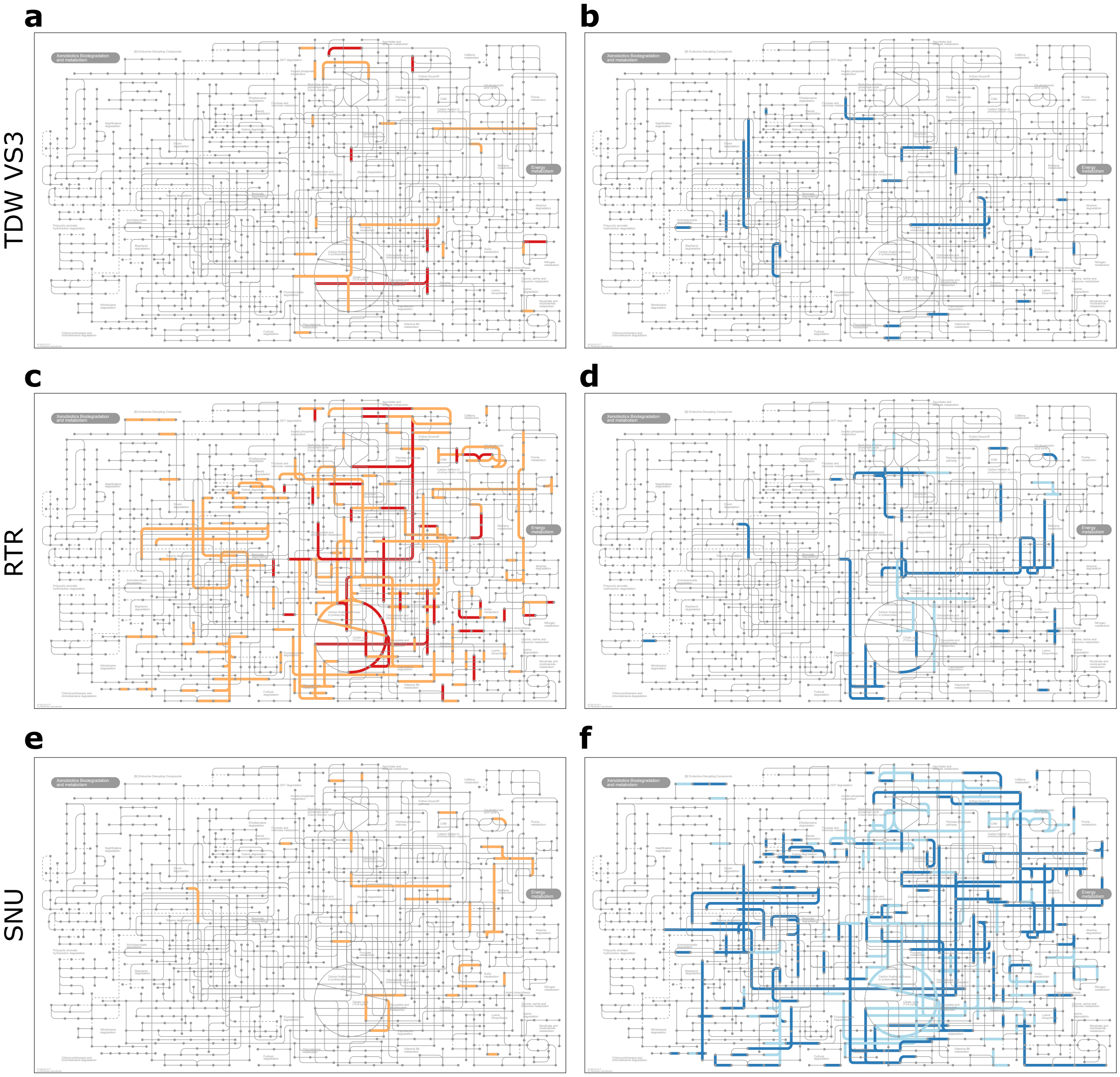
a
b
TDW VS3
c
d
RTR
e
f
SNU

Fig. S6 Number of potential enzyme classification (EC) per KEGG modules over/under-represented in bacterial OTU linked to *Medicago truncatula* phenotype

OTUs that were major predictors of the plant ecophysiological traits (i.e. total biomass, root to total ratio and specific nitrogen uptake), were first identified with three regression random forest (RF) analyses. To test whether some functions were potentially more or less abundant for the candidate OTU compared to the rest of the bacterial communities, we considered the extended list of OTUs that were significantly correlated to the candidate OTUs identified by the random forests. These lists of OTUs were then divided into two: OTUs positively and negatively correlated to each of the plant phenotypic variables. A two-sided t-test using the function getPvalues of topGO R package (Alexa & Rahnenfuhrer, Jorg, 2016) was applied to the EC_predicted.tsv output file from PICRUSt2, for which both rare and non-well characterized OTU (NSTI>2) were previously removed, in order to identify enzyme classifications (EC) that were potentially differentially abundant between the extended candidate OTUs list and the rest of the non-rare OTUs. Finally, KEGG modules associated to the significantly differentially abundant EC were defined using the EC KEGG correspondence table downloaded from https://www.genome.jp/kegg-bin/get_htext#B3 (version of the October 14, 2020).

Bars colored i) in dark red represent EC number more abundant in the extended candidate OTUs list than in the rest of the OTUs for the OTUs that are positively corelated to the plant ecophysiological trait, ii) in light red represent EC number less abundant in the extended candidate OTUs list than in the rest of the OTUs for the OTUs that are negatively corelated to the ecophysiological trait, iii) in dark blue represent EC number less abundant in the extended candidate OTUs list than in the rest of the OTUs for the OTUs that are positively corelated to the ecophysiological trait, and iv) in light blue represent EC number more abundant in the extended candidate OTUs list than in the rest of the OTUs for the OTUs that are negatively corelated to the ecophysiological trait.

Abbreviations: TDW: Total Dry Weight (g); VS3: Vegetative developmental Stage 3 (848 degree-days after sowing or 49 days after sowing); RTR: Root to Total biomass Ratio, SNU: plant-Specific Nitrogen Uptake (g N g-1 of belowground dry biomass day-1).

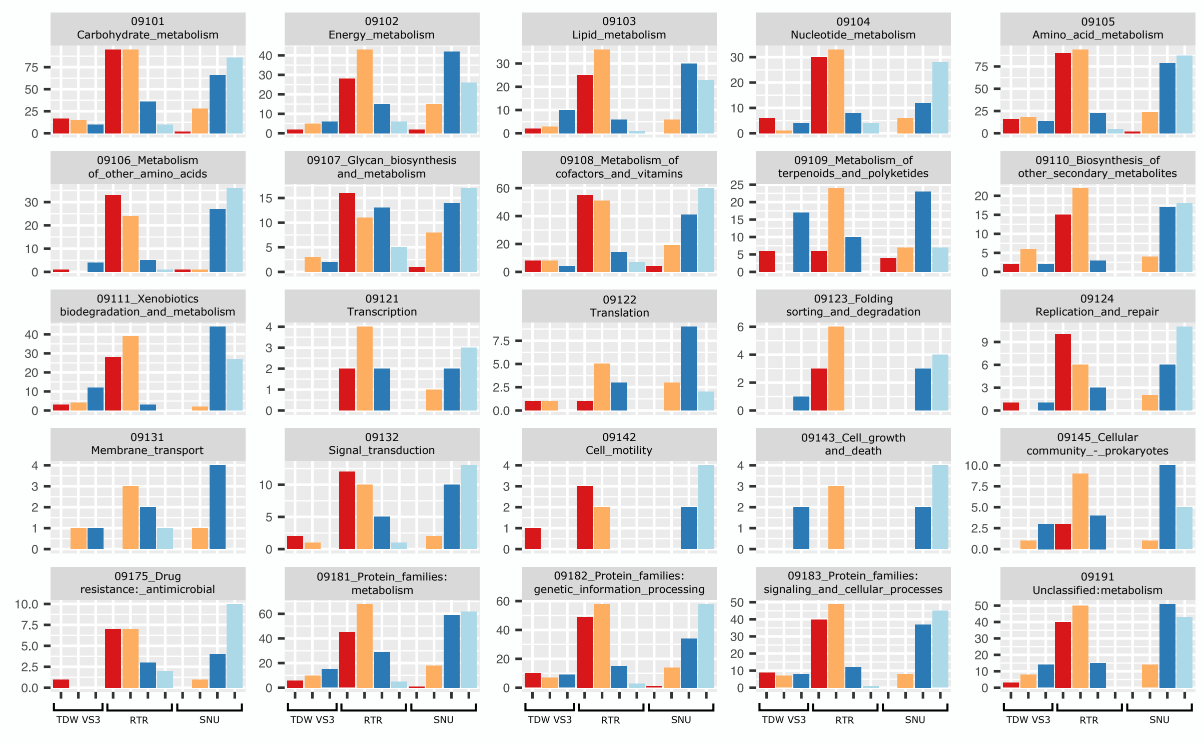
09101
Carbohydrate_metabolism
09102
Energy_metabolism
09103
Lipid_metabolism
09104
Nucleotide_metabolism
09105
Amino_acid_metabolism
09106_Metabolism
of_other_amino_acids
09107_Glycan_biosynthesis
and_metabolism
09108_Metabolism_of
cofactors_and_vitamins
09109_Metabolism_of
terpenoids_and_polyketides
09110_Biosynthesis_of
other_secondary_metabolites
09111_Xenobiotics
biodegradation_and_metabolism
09121
Transcription
09122
Translation
09123_Folding
sorting_and_degradation
09124
Replication_and_repair
09131
Membrane_transport
09132
Signal_transduction
09142
Cell_motility
09143_Cell_growth
and_death
09145_Cellular
community_-_prokaryotes
09175_Drug
resistance:_antimicrobial
09181_Protein_families:
metabolism
09182_Protein_families:
genetic_information_processing
09183_Protein_families:
signaling_and_cellular_processes
09191
Unclassified:metabolism
TDW VS3
RTR
SNU

Fig. S7 Coefficients of variation for the heritable OTUs

Boxplot showing the coefficient of variation for each heritable OTU. Coefficients of variation were calculated for each heritable OTU and each plant genotype as the ratio of the standard deviation to the average for the three replicates (n=155). The black line represents the median value. The lower and upper hinges correspond to the first and third quartiles. The upper and lower whiskers extend from the hinge to the largest and smallest value, respectively (no further than 1.5 time the inter-quartile range). Data beyond the end of the whiskers are plotted individually as black dots.

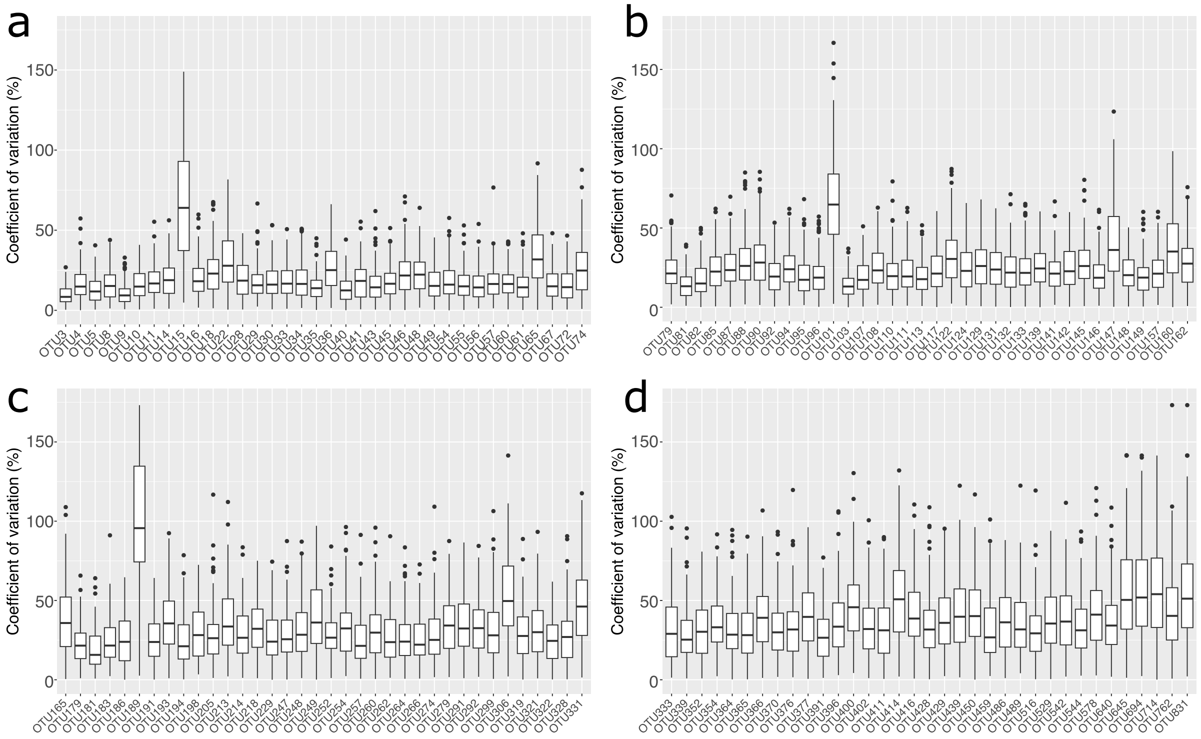

New Phytologist

Fig. S8 Genetic variation within *M. truncatula* shapes the composition of the most heavily sequenced OTUs of the rhizosphere bacterial communities

(**a**) After Hellinger transformation, Principal Component Analyses (PCA) were performed on the filtered and normalized occurrence table (n=435, 155 plant genotypes and their replicates) for all the OTUs, the 50%, 20%, 15%, 10%, 5%, 4%, 3%, 2%, and 1% most heavily sequenced OTUs. Significance of the regression of the plant genotype variable on ordination axes of the PCA was tested using the Vegan enfit function and 999 permutations. P-value= 0.33, 0.27, 0.12, 0.051, 0.027, 0.005 and 0.001 for all the OTU (i.e. top 100%), the top 50%, 20%, 15%, 10%, 5% and 1%, respectively.

(**b**) Percentage of the total sequences represented by the number of bacterial OTUs sorted from the most abundant to the less abundant ones.

(**c**) Principal Component Analyses (PCA) on the top 10% most heavily sequenced OTU. Dots in black represent the samples (n=435).

(**d**) To reveal plant genes involved in the structure of the bacterial community of *M. truncatula*, GWAS was conducted on the first two principal component of the Principal Component Analysis including only the top 10% most heavily sequenced OTU, after Hellinger transformation. SNP*OTU associations were selected based on a P-value threshold of 10-6.

Abbreviations: PC: principal component.

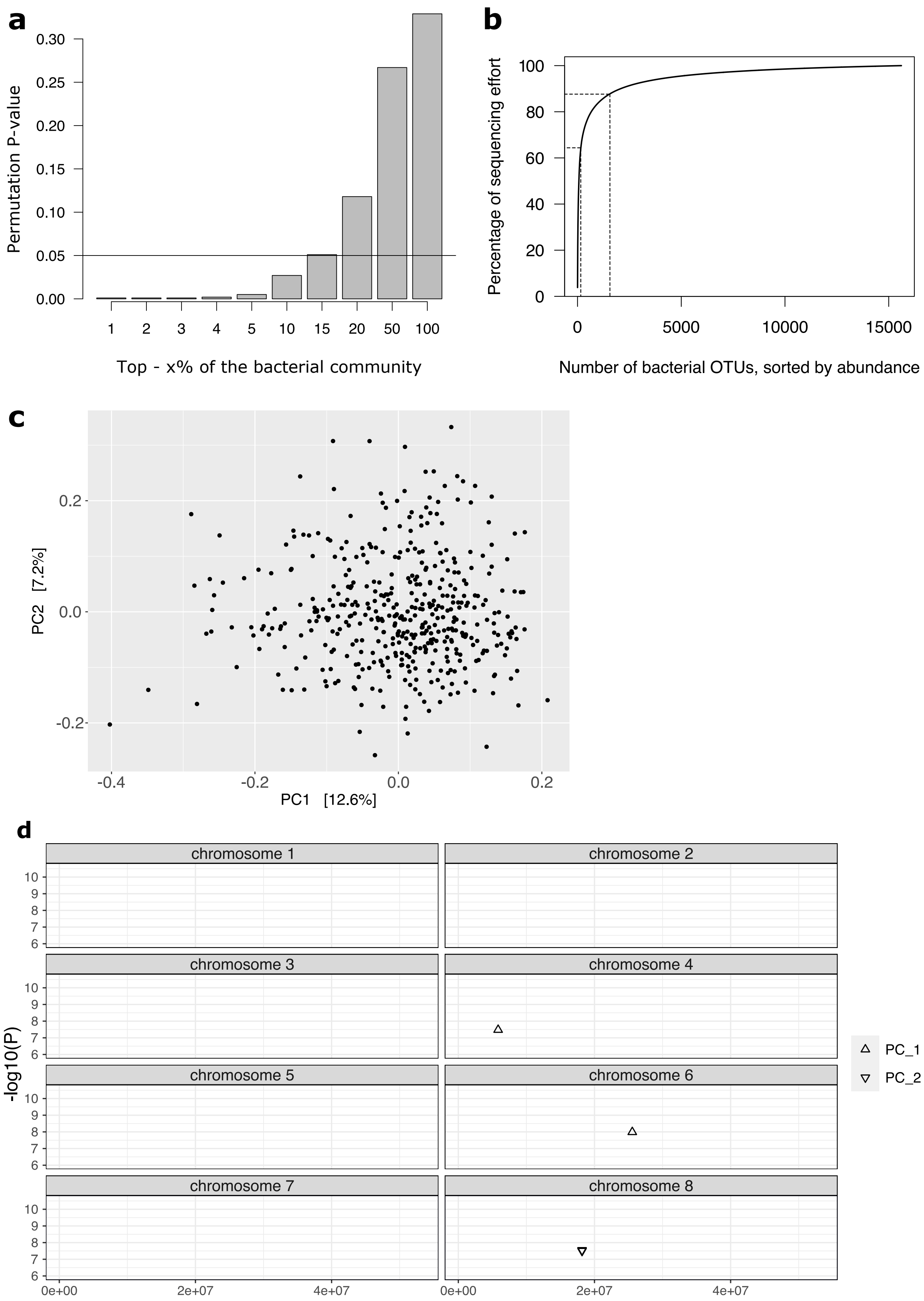
a
Permutation P-value
0.30
0.25
0.20
0.15
0.10
0.05
0.00
1 2 3 4 5 10 15 20 50 100
Top - x% of the bacterial community
b
Percentage of sequencing effort
100
80
60
40
20
0
0 5000 10000 15000
Number of bacterial OTUs, sorted by abundance
c
PC2 [7.2%]
0.2
0.0
-0.2
-0.4 -0.2 0.0 0.2
PC1 [12.6%]
d
chromosome 1
chromosome 2
chromosome 3
chromosome 4
chromosome 5
chromosome 6
chromosome 7
chromosome 8
-log10(P)
10 9 8 7 6
0e+00 2e+07 4e+07
PC_1
PC_2

Table S1 Plant genotype list and their geographical origin

Table S2 Plant position in glasshouse

Abbreviations: VS1: Vegetative developmental Stage 1 (488 degree-days or 28 days after sowing); VS2: Vegetative developmental Stage 2 (734 degree-days or 42 days after sowing); VS3: Vegetative developmental Stage 3 (848 degree-days or 49 days after sowing).

Table S3 Acronym list

Table S4 Plant phenotypic variables measured and calculated for the core collection of *M. truncatula* (n=155)

Averages were calculated for each variable using the mean values per plant genotype.

The five ecophysiological groups of plant genotypes were defined using the hclust "ward.D2" linkage method in R software based on the ecophysiological dataset including only the non-highly correlated variables (|Spearman correlation| < 0.6; LA VS1, TDW VS1, RTR VS1, NR VS1, TDW VS2, RTR VS2, NR VS2, LA VS3, TDW VS3, NNI VS3, RUE, RTR, SNU). The genetic groups are the one used in GWAS (based on Bonhomme et al., 2014).

Abbreviations: LA: Leaf Area (cm^2) ; SDW: Shoot Dry Weight (g); RDW: Root Dry Weight (g); TDW: Total Dry Weight (g); NC: Nitrogen content (g); NR: Nitrogen Ratio (%); NNI: Nitrogen Nutrition Index; NLA: conversion factor of Nitrogen to Leaf Area (cm^2 of leaf g-1 of N); RUE: Radiation Use Efficiency (g of total dry biomass MJ-1 of intercepted PAR); RTR: Root to Total biomass Ratio, SNU: plant-Specific Nitrogen Uptake (g N g-1 of belowground dry biomass day-1); VS1: Vegetative developmental Stage 1 (488 degree-days or 28 days after sowing); VS2: Vegetative developmental Stage 2 (734 degree-days or 42 days after sowing); VS3: Vegetative developmental Stage 3 (848 degree-days or 49 days after sowing).

Table S5 Estimation of the heritability of the ecophysiological parameters

Heritability (h2) estimates the proportion of phenotypic variance that is due to genetic variance. Genetic variance was estimated fitting the linear mixed model Yij = Gi + eij, where Yij is the vector of normalized count for each OTU for each genotype i and random repeat k using the R package lme4 version 1.1-31 (Bates et al., 2015)). Then, h2 was calculated as genetic variance (varG) divided by the sum of genetic variance and the error variance (varG + varerror/n), where n=3 is the total number of replicates in our random design. We considered as heritable a variable with significant non-zero genetic variance (Pvalue of the likelihood ratio test for the genetic effect lower than 0.05) and h2 higher than 0.25.

Table S6 GWAS output for the ecophysiological parameters

Single Nucleotide Polymorphism (SNP) were selected for each ecophysiological trait based on the presence of at least 3 SNP at a P-value threshold of 10-6 within a 30 kB interval.
Abbreviations: CHR: chromosome; BP: position; miss: number of missing values in genotype data; alt: alternative allele; ref: reference allele; af: minor allele frequency; P: p_lrt value; effect: SNP effect, type of variant; codon_change: induced by SNP; AA_change: induced by SNP; NOTE: gene annotation.

Table S7 List of Gene IDs corresponding to the significant SNPs in the GWAS for each ecophysiological traits and their corresponding annotation, GO id and GO term

Table S8 Richness, alpha- and beta-diversity indices of the rhizosphere bacterial communities associated with the *Medicago truncatula* core collection

Observed richness, Chao 1, Shannon, evenness, inverse Simpson and Bray-Curtis dissimilarities were calculated using the vegan package, while Sørensen dissimilarities were calculated using the betapart package. Averages and standard deviations were calculated for each variable using the mean values per plant genotype or per distance values for the beta-diversity indices. Significance of Kruskal-Wallis tests: ns, * and ** indicate not significant and significant levels at 0.05 and 0.01, respectively. Letters with different labels indicate significant differences (P < 0.05) for Dunn tests. The five ecophysiological groups of plant genotypes (E1-E5) were defined using the hclust "ward.D2" linkage method in R software based on the ecophysiological dataset including only the thirteen non-highly correlated variables (|Spearman correlation| < 0.6). Abbreviations: E for Ecophysiological; beta.SIM represents the value of the turnover component, measured as Sørensen dissimilarity; beta.SNE represents the value of the nestedness component, measured as nestedness-resultant fraction of Sørensen dissimilarity (beta.multi function of the betapart R package).

	**Average ± sd**	**Kruskal-Wallis tests**						**Average and Dunn tests**				
		**Plant genotype**			**Ecophysiological group**							
		Degrees of freedom	Chi-square	p-value	Degrees of freedom	Chi-square	p-value	Group E1	Group E2	Group E3	Group E4	Group E5
Richness	7656 ± 632	154	160.31	0.35 ns	4	9.4899	0.05 *	7530 a	7595 a	7921 a	7481 a	7813 a
Chao 1	18476 ± 1701	154	137.03	0.83 ns	4	7.8428	0.10 ns	18162 a	18328 a	19205 a	17964 a	18842 a
Shannon	6.4 ± 0.1	154	199.69	0.008 **	4	14.807	0.005 **	6.33 b	6.34 ab	6.40 a	6.34 ab	6.38 ab
Evenness	0.74 ± 0.01	154	213.23	0.001 **	4	17.078	0.002 **	0.74 b	0.74 b	0.75 a	0.74 b	0.74 ab
Inverse Simpson	129 ± 11	154	172.91	0.14 ns	4	9.2733	0.05 ns	124 a	128 a	132 a	129 a	132 a
Bray-Curtis dissimilarity	0.25 ± 0.04	-	-	-	-	-	-	-	-	-	-	-
Whittaker	0.60 ± 0.01	-	-	-	-	-	-	-	-	-	-	-
Sorensen	0.43 ± 0.02	-	-	-	-	-	-	-	-	-	-	-
beta.SIM	0.40 ± 0.03	-	-	-	-	-	-	-	-	-	-	-
beta.SNE	0.03 ± 0.02	-	-	-	-	-	-	-	-	-	-	-

Table S9 Filtered and normalized occurrence table including all samples (n=435) used in alpha- and beta-diversity analyses, bacterial composition, and functional prediction

Table S10 Taxonomic affiliation, relative abundance, and properties in the co-occurrence network of the 150 OTU found significantly correlated in the co-occurrence network analysis

Table S11 PERMANOVA results based on Bray-Curtis distances

PERMANOVA were calculated using the vegan package and adonis function. Significance of PERMANOVA tests: * and *** indicate significant levels at 0.05 and 0.001, respectively.

	Degrees of freedom	Sums of squares	Mean square	F.Model	R^2	Pr (>F)
Hapmap_id	154	5.3190	0.034539	1.1914	0.39588	1e-04 ***
Residuals	280	8.1169	0.028989		0.60412	
Total	434	13.4359			1.00000	
Ecophysiological_group	4	0.1593	0.039827	1.2949	0.01263	0.0115 *
Residuals	405	12.4560	0.030756		0.98737	
Total	409	12.6153			1.00000	

Table S12 Plant genotype effect on bacterial composition at Phylum and Class levels and on KEGG categories

Significance of the Kruskal-Wallis tests: ns, *, ** and *** indicate not significant and significant levels at 0.05, 0.01 and 0.001, respectively.

Table S13 Redundancy analysis on microbial data and ecophysiological for the core collection of *M. truncatula*

Redundancy analyze (RDA) was performed using the vegan package and rda function. The model used was rda(formula = data_microbio ~ LA_VS3 + TDW_VS3 + NNI_VS3 + RTR + SNU + RUE + TDW_VS2 + RTR_VS2 + NR_VS2 + LA_VS1 + TDW_VS1 + RTR_VS1 + NR_VS1, data = sample_metadata).

Both the significance of the model and the marginal effects in the model were tested using an ANOVA-like permutation test with 9999 permutations using anova.cca function and parameter by=”margin” for the marginal effects.

Significance of RDA tests: ns, * and *** indicate not significant and significant levels at 0.05 and 0.001, respectively.

Abbreviations: LA: Leaf Area ; TDW: Total Dry Weight ; NNI: Nitrogen Nutritional Index ; RTR: Root to Total biomass Ratio ; SNU: Specific Nitrogen Uptake ; RUE: Radiation Use Efficiency ; NR: Nitrogen Ratio ; VS: Vegetative Stage.

	RDA			ANOVA.CCA			
	Inertia	Proportion	Rank	Degrees of freedom	Variance	F	Pr (>F)
Total	0.053594	1.000000					
Constrained	0.005506	0.102730	13				
Unconstrained	0.048088	0.897270	132				
**by=NULL**							
Model				13	0.005506	1.1625	1e-04 ***
Residuals				132	0.048088		
**by=”margin”**							
LA_VS3				1	0.000390	1.0716	0.2209
TDW_VS3				1	0.000371	1.0172	0.3598
NNI_VS3				1	0.000343	0.9416	0.6881
RTR				1	0.000455	1.2492	0.0229 *
SNU				1	0.000362	0.9938	0.4517
RUE				1	0.000345	0.9458	0.6536
TDW_VS2				1	0.000428	1.1747	0.0552
RTR_VS2				1	0.000324	0.8899	0.9065
NR_VS2				1	0.000476	1.3069	0.0111 *
LA_VS1				1	0.000389	1.0689	0.2111
TDW_VS1				1	0.000340	0.9329	0.7329
RTR_VS1				1	0.000374	1.0260	0.3297
NR_VS1				1	0.000352	0.9667	0.5730
Residual				132	0.048088		

Table S14 List of the candidate OTU, which are found as major predictor of three plant phenotypic variables and their significantly correlated OTU

Abbreviations: TDW: Total Dry Weight (g); RTR: Root to Total biomass Ratio, SNU: plant-Specific Nitrogen Uptake (g N g-1 of belowground dry biomass day-1); RF: random forest; VIPlist: major OTU predictors for the plant phenotypic variable analyzed; ExtendedVIPlist: major OTU predictors for the plant phenotypic variable analyzed and their significantly correlated OTU, but without OTU with a nearest-sequenced taxon index (NSTI) value superior to 2.

Table S15 Potential enzyme classification that are significantly more and less abundant for the OTU positively and negatively linked to the three plant phenotypic variables analyzed in random forest in comparison to the rest of the bacterial OTU and their associated KEGG categories and pathways

Table S16 Estimation of the heritability for the 900 most abundant OTUs

Heritability (h2) estimates the proportion of phenotypic variance that is due to genetic variance. Genetic variance was estimated fitting the linear mixed model Yij = Gi + eij, where Yij is the vector of normalized count for each OTU for each genotype i and random repeat k using the R package lme4 version 1.1-31 (Bates et al., 2015)). Then, h2 was calculated as genetic variance (varG) divided by the sum of genetic variance and the error variance (varG + varerror/n), where n=3 is the total number of replicates in our random design. We considered as heritable a variable with significant non-zero genetic variance (Pvalue of the likelihood ratio test for the genetic effect lower than 0.05) and h2 higher than 0.25.

Table S17 Global GWAS output for the abundance of the bacterial OTUs (OTUs predicting plant ecophysiological traits, OTUs in the cooccurrence network and the top 157 most abundant OTUs) and for the two first principal component axes of the ordination plot representing the bacterial community (using the top 10% more abundant OTU)).

Single Nucleotide Polymorphism (SNP) were selected for each OTU based on the presence of at least 3 SNP at a P-value threshold of 10-6within a 30 kB interval.
Abbreviations: CHR: chromosome; BP: position; miss: number of missing values in genotype data; alt: alternative allele; ref: reference allele; af: minor allele frequency; P: p_lrt value; effect: SNP effect, type of variant; codon_change: induced by SNP; AA_change: induced by SNP; NOTE: gene annotation.

Table S18 List of Gene IDs corresponding to the significant SNPs in the GWAS for each analysis of VIP OTU group predicting ecophysiological traits and their corresponding annotation, GO id and GO term

Table S19 List of Gene IDs corresponding to the significant SNPs in the GWAS for each OTU group (cooccurrence network and top 157 most abundant ones), their corresponding annotation, GO id and GO term, and topGO output

Notes S1 Zip file containing data sets and R scripts used to produce the analyses and figures

References

Alexa A, Rahnenfuhrer, Jorg. **2016**. topGO: Enrichment Analysis for Gene Ontology. R package version 2.24.0. *Bioconductor*.

Baselga A. **2010**. Partitioning the turnover and nestedness components of beta diversity. *Global Ecology and Biogeography*: 134–143.

Baselga A, Orme CDL. **2012**. betapart : an R package for the study of beta diversity: *Betapart package*. *Methods in Ecology and Evolution* **3**: 808–812.

Bates D, Mächler M, Bolker B, Walker S. **2015**. Fitting Linear Mixed-Effects Models Using **lme4**. *Journal of Statistical Software* **67**.

Benjamini Y, Hochberg Y. **1995**. Controlling the False Discovery Rate: A Practical and Powerful Approach to Multiple Testing. *Journal of the Royal Statistical Society. Series B (Methodological)* **57**: 289–300.

Bonhomme M, André O, Badis Y, Ronfort J, Burgarella C, Chantret N, Prosperi J, Briskine R, Mudge J, Debéllé F, *et al.* 2014. High-density genome-wide association mapping implicates an F -box encoding gene in *M edicago truncatula* resistance to *A phanomyces euteiches* . *New Phytologist* **201**: 1328–1342.

Breiman L. **2001**. Random Forests. *Machine Learning* **45**: 5–32.

Csardi G, Nepusz T. **2006**. The igraph software package for complex network research.

Deng Y, Umbach AK, Neufeld JD. **2024**. Nonparametric richness estimators Chao1 and ACE must not be used with amplicon sequence variant data. *The ISME Journal* **18**: wrae106.

Dillies M-A, Rau A, Aubert J, Hennequet-Antier C, Jeanmougin M, Servant N, Keime C, Marot G, Castel D, Estelle J, *et al.* 2013. A comprehensive evaluation of normalization methods for Illumina high-throughput RNA sequencing data analysis. *Briefings in Bioinformatics* **14**: 671–683.

Djemiel C, Dequiedt S, Karimi B, Cottin A, Girier T, El Djoudi Y, Wincker P, Lelièvre M, Mondy S, Chemidlin Prévost-Bouré N, *et al.* 2020. BIOCOM-PIPE: a new user-friendly metabarcoding pipeline for the characterization of microbial diversity from 16S, 18S and 23S rRNA gene amplicons. *BMC Bioinformatics* **21**: 492.

Douglas GM, Maffei VJ, Zaneveld JR, Yurgel SN, Brown JR, Taylor CM, Huttenhower C, Langille MGI. **2020**. PICRUSt2 for prediction of metagenome functions. *Nature Biotechnology* **38**: 685–

688.

Friedman J, Alm EJ. **2012**. Inferring correlation networks from genomic survey data. *PLoS computational biology* **8**: e1002687.

Gastal F, Lemaire G. **2002**. N uptake and distribution in crops: an agronomical and ecophysiological perspective. *Journal of Experimental Botany* **53**: 789–799.

Horton MW, Bodenhausen N, Beilsmith K, Meng D, Muegge BD, Subramanian S, Vetter MM, Vilhjálmsson BJ, Nordborg M, Gordon JI, *et al.* 2014. Genome-wide association study of Arabidopsis thaliana leaf microbial community. *Nature Communications* **5**: 5320.

Langfelder P, Horvath S. **2008**. WGCNA: an R package for weighted correlation network analysis. *BMC Bioinformatics* **9**: 559.

Langille MGI, Zaneveld J, Caporaso JG, McDonald D, Knights D, Reyes JA, Clemente JC, Burkepile DE, Vega Thurber RL, Knight R, *et al.* 2013. Predictive functional profiling of microbial communities using 16S rRNA marker gene sequences. *Nature Biotechnology* **31**: 814–821.

Le Signor C, Aimé D, Bordat A, Belghazi M, Labas V, Gouzy J, Young ND, Prosperi J-M, Leprince O, Thompson RD, *et al.* 2017. Genome-wide association studies with proteomics data reveal genes important for synthesis, transport and packaging of globulins in legume seeds. *The New Phytologist* **214**: 1597–1613.

Moreau D, Burstin J, Aubert G, Huguet T, Ben C, Prosperi J-M, Salon C, Munier-Jolain N. **2012**. Using a physiological framework for improving the detection of quantitative trait loci related to nitrogen nutrition in Medicago truncatula. *TAG. Theoretical and applied genetics. Theoretische und angewandte Genetik* **124**: 755–768.

Moreau D, Schneider C, Huguet T, Salon C, Munier-Jolain N. **2009**. Can differences of nitrogen nutrition level among *Medicago truncatula* genotypes be assessed non-destructively? Probing with a recombinant inbred lines population: Probing with a recombinant inbred lines population. *Plant Signaling & Behavior* **4**: 30–32.

Moreau D, Voisin A-S, Salon C, Munier-Jolain N. **2008**. The model symbiotic association between Medicago truncatula cv. Jemalong and Rhizobium meliloti strain 2011 leads to N-stressed plants when symbiotic N2 fixation is the main N source for plant growth. *Journal of Experimental Botany* **59**: 3509–3522.

Mougel C, Offre P, Ranjard L, Corberand T, Gamalero E, Robin C, Lemanceau P. **2006**. Dynamic of the genetic structure of bacterial and fungal communities at different developmental stages of Medicago truncatula Gaertn. cv. Jemalong line J5. *New Phytologist* **170**: 165–175.

Offre P, Pivato B, Siblot S, Gamalero E, Corberand T, Lemanceau P, Mougel C. **2007**. Identification of bacterial groups preferentially associated with mycorrhizal roots of Medicago truncatula. *Applied and Environmental Microbiology* **73**: 913–921.

Oksanen J, Blanchet G, Friendly M, Kindt R, Legendre P, McGlinn D, Minchin PR, O'Hara RB, Simpson GL, Solymos P, *et al.* 2020. vegan: Community Ecology Package. R package version 2.5-7.

R Development Core Team. **2014**. R: A Language and Environment for Statistical Computing. *R Foundation for Statistical Computing, Vienna, Austria*.

RStudio Team. **2020**. RStudio: Integrated Development for R. RStudio. *PBC, Boston, MA*.

Schloss PD, Westcott SL. **2011**. Assessing and improving methods used in operational taxonomic unit-based approaches for 16S rRNA gene sequence analysis. *Applied and Environmental Microbiology* **77**: 3219–3226.

Shannon P, Markiel A, Ozier O, Baliga NS, Wang JT, Ramage D, Amin N, Schwikowski B, Ideker T. **2003**. Cytoscape: a software environment for integrated models of biomolecular interaction networks. *Genome Research* **13**: 2498–2504.

Shi L, Westerhuis JA, Rosén J, Landberg R, Brunius C. **2019**. Variable selection and validation in multivariate modelling. *Bioinformatics (Oxford, England)* **35**: 972–980.

Stanton-Geddes J, Paape T, Epstein B, Briskine R, Yoder J, Mudge J, Bharti AK, Farmer AD, Zhou P, Denny R, *et al.* 2013. Candidate Genes and Genetic Architecture of Symbiotic and Agronomic Traits Revealed by Whole-Genome, Sequence-Based Association Genetics in Medicago truncatula (L Lukens, Ed.). *PLoS ONE* **8**: e65688.

Tang H, Krishnakumar V, Bidwell S, Rosen B, Chan A, Zhou S, Gentzbittel L, Childs KL, Yandell M, Gundlach H, *et al.* 2014. An improved genome release (version Mt4.0) for the model legume Medicago truncatula.

Terrat S, Christen R, Dequiedt S, Lelièvre M, Nowak V, Regnier T, Bachar D, Plassart P, Wincker P, Jolivet C, *et al.* 2012. Molecular biomass and MetaTaxogenomic assessment of soil microbial

communities as influenced by soil DNA extraction procedure: Soil DNA extraction impact on bacterial diversity. *Microbial Biotechnology* **5**: 135–141.

Terrat S, Plassart P, Bourgeois E, Ferreira S, Dequiedt S, Adele-Dit-De-Renseville N, Lemanceau P, Bispo A, Chabbi A, Maron P-A, *et al.* 2015. Meta-barcoded evaluation of the ISO standard 11063 DNA extraction procedure to characterize soil bacterial and fungal community diversity and composition. *Microbial Biotechnology* **8**: 131–142.

Větrovský T, Baldrian P. **2013**. The variability of the 16S rRNA gene in bacterial genomes and its consequences for bacterial community analyses. *PloS One* **8**: e57923.

Weiss S, Xu ZZ, Peddada S, Amir A, Bittinger K, Gonzalez A, Lozupone C, Zaneveld JR, Vázquez-Baeza Y, Birmingham A, *et al.* 2017. Normalization and microbial differential abundance strategies depend upon data characteristics. *Microbiome* **5**: 27.

Zancarini A, Mougel C, Terrat S, Salon C, Munier-Jolain N. **2013**. Combining ecophysiological and microbial ecological approaches to study the relationship between Medicago truncatula genotypes and their associated rhizosphere bacterial communities. *Plant and Soil* **365**: 183–199.

Zhou X, Stephens M. **2012**. Genome-wide efficient mixed-model analysis for association studies. *Nature Genetics* **44**: 821–824.
