# Supplementary material for: Medicago truncatula genotype drives the plant nutritional strategy and its associated rhizosphere bacterial communities
Source: New Phytol. 2024 Nov 28;245(2):767–84. doi: 10.1111/nph.20272 (PMC11655437; doi:10.1111/nph.20272)
Supplement: Supplementary file 3 — Table S1 Plant genotype list and their geographical origin. Table S2 Plant position in glasshouse. Table S3 Acronym list. Table S4 Plant phenotypic variables measured and calculated for each genotype of the core collection of Medicago truncatula. Table S5 Estimation of the heritability of the ecophysiological parameters. Table S6 GWAS output for the ecophysiological parameters. Table S7 List of Gene IDs corresponding to the significant SNPs in the GWAS for each ecophysiological traits and their corresponding annotation, GO id and GO term. Table S8 Richness, alpha‐ and beta‐diversity indices of the rhizosphere bacterial communities associated with the Medicago truncatula core collection. Table S9 Filtered and normalized occurrence table including all samples (n = 435) used in alpha‐ and beta‐diversity analyses, bacterial composition, and functional prediction. Table S10 Taxonomic affiliation, relative abundance, and properties in the co‐occurrence network of the 150 OTU found significantly correlated in the co‐occurrence network analysis. Table S11 PERMANOVA results based on Bray‐Curtis distances. Table S12 Plant genotype effect on bacterial composition at Phylum and Class levels and on KEGG categories. Table S13 Redundancy analysis on microbial data and ecophysiological for the core collection of Medicago truncatula. Table S14 List of the candidate OTU, which are found as major predictor of three plant phenotypic variables and their significantly correlated OTU. Table S15 Potential enzyme classification that are significantly more and less abundant for the OTU positively and negatively linked to the three plant phenotypic variables analyzed in random forest in comparison to the rest of the bacterial OTU and their associated KEGG categories and pathways. Table S16 Estimation of the heritability for the 900 most abundant OTUs. Table S17 Global GWAS output for the abundance of the bacterial OTUs (OTUs predicting plant ecophysiological traits, OTUs in the co‐occurrence [file NPH-245-767-s002.zip › Table_S13_rdaStat.docx]

**Table supplemental S3:** Redundancy analysis on microbial data and ecophysiological for the core collection of *M. truncatula*

|  |  | **RDA** |  |  | **ANOVA.CCA** | |  |
| --- | --- | --- | --- | --- | --- | --- | --- |
|  | Inertia | Proportion | Rank | Degrees of freedom | Variance | F | Pr (>F) |
| Total | 0.053594 | 1.000000 |  |  |  |  |  |
| Constrained | 0.005506 | 0.102730 | 13 |  |  |  |  |
| Unconstrained | 0.048088 | 0.897270 | 132 |  |  |  |  |
|  |  |  |  |  |  |  |  |
| **by=NULL** |  |  |  |  |  |  |  |
| Model |  |  |  | 13 | 0.005506 | 1.1625 | 1e-04 *** |
| Residuals |  |  |  | 132 | 0.048088 |  |  |
|  |  |  |  |  |  |  |  |
| **by=”term”** |  |  |  |  |  |  |  |
| LA_VS3 |  |  |  | 1 | 0.000390 | 1.0716 | 0.2209 |
| TDW_VS3 |  |  |  | 1 | 0.000371 | 1.0172 | 0.3598 |
| NNI_VS3 |  |  |  | 1 | 0.000343 | 0.9416 | 0.6881 |
| RTR |  |  |  | 1 | 0.000455 | 1.2492 | 0.0229 * |
| SNU |  |  |  | 1 | 0.000362 | 0.9938 | 0.4517 |
| RUE |  |  |  | 1 | 0.000345 | 0.9458 | 0.6536 |
| TDW_VS2 |  |  |  | 1 | 0.000428 | 1.1747 | 0.0552 |
| RTR_VS2 |  |  |  | 1 | 0.000324 | 0.8899 | 0.9065 |
| NR_VS2 |  |  |  | 1 | 0.000476 | 1.3069 | 0.0111 * |
| LA_VS1 |  |  |  | 1 | 0.000389 | 1.0689 | 0.2111 |
| TDW_VS1 |  |  |  | 1 | 0.000340 | 0.9329 | 0.7329 |
| RTR_VS1 |  |  |  | 1 | 0.000374 | 1.0260 | 0.3297 |
| NR_VS1 |  |  |  | 1 | 0.000352 | 0.9667 | 0.5730 |
| Residual |  |  |  | 132 | 0.048088 |  |  |
|  |  |  |  |  |  |  |  |

All analyses were performed using the statistical software R 4.1.1 within the RStudio Integrated Development Environment 1.4.1717.

Redundancy analyze (RDA) was performed using the vegan package and rda function. The model used was rda(formula = data_microbio ~ LA_VS3 + TDW_VS3 + NNI_VS3 + RTR + SNU + RUE + TDW_VS2 + RTR_VS2 + NR_VS2 + LA_VS1 + TDW_VS1 + RTR_VS1 + NR_VS1, data = sample_metadata).

Both the significance of the model and the marginal effects in the model were tested using an ANOVA-like permutation test with 9999 permutations using anova.cca function and parameter by=”margin” for the marginal effects.

Significance of RDA tests: ns, * and *** indicate not significant and significant levels at 0.05 and 0.001, respectively.

Abbreviations: LA Leaf Area ; TDW Total Dry Weight ; NNI Nitrogen Nutritional Index ; RTR Root:Total biomass Ratio ; SNU Specific Nitrogen Uptake ; RUE Radiation Use Efficiency ; NR Nitrogen Ratio ; VS Vegetative Stage.
